# Ex Vivo Functional Benchmarking of Hyaluronan-Based Osteoarthritis Viscosupplement Products: Comprehensive Assessment of Rheological, Lubricative, Adhesive, and Stability Attributes

**DOI:** 10.3390/gels9100808

**Published:** 2023-10-09

**Authors:** Alexandre Porcello, Farid Hadjab, Maryam Ajouaou, Virginie Philippe, Robin Martin, Philippe Abdel-Sayed, Nathalie Hirt-Burri, Corinne Scaletta, Wassim Raffoul, Lee Ann Applegate, Eric Allémann, Olivier Jordan, Alexis Laurent

**Affiliations:** 1 School of Pharmaceutical Sciences, University of Geneva, CH-1206 Geneva, Switzerland; maryam.ajouaou@etu.unige.ch (M.A.); eric.allemann@unige.ch (E.A.); olivier.jordan@unige.ch (O.J.); 2 Institute of Pharmaceutical Sciences of Western Switzerland, University of Geneva, CH-1206 Geneva, Switzerland; 3 Development Department, Albomed GmbH, D-90592 Schwarzenbruck, Germany; f.hadjab@albomed.eu; 4 Regenerative Therapy Unit, Lausanne University Hospital, University of Lausanne, CH-1066 Epalinges, Switzerland; virginie.philippe@chuv.ch (V.P.); philippe.abdel-sayed@chuv.ch (P.A.-S.); nathalie.burri@chuv.ch (N.H.-B.); corinne.scaletta@chuv.ch (C.S.); wassim.raffoul@chuv.ch (W.R.); lee.laurent-applegate@chuv.ch (L.A.A.); 5 Orthopedics and Traumatology Service, Lausanne University Hospital, University of Lausanne, CH-1011 Lausanne, Switzerland; robin.martin@chuv.ch; 6 STI School of Engineering, Federal Polytechnic School of Lausanne, CH-1015 Lausanne, Switzerland; 7 Center for Applied Biotechnology and Molecular Medicine, University of Zurich, CH-8057 Zurich, Switzerland; 8 Oxford OSCAR Suzhou Center, Oxford University, Suzhou 215123, China; 9 Manufacturing Department, LAM Biotechnologies SA, CH-1066 Epalinges, Switzerland

**Keywords:** adhesivity, antioxidants, functional attributes, hyaluronic acid, hydrogel stability, lubrication, osteoarthritis, rheology, tribology, viscosupplementation

## Abstract

While many injectable viscosupplementation products are available for osteoarthritis (OA) management, multiple hydrogel functional attributes may be further optimized for efficacy enhancement. The objective of this study was to functionally benchmark four commercially available hyaluronan-based viscosupplements (Ostenil, Ostenil Plus, Synvisc, and Innoryos), focusing on critical (rheological, lubricative, adhesive, and stability) attributes. Therefore, in vitro and ex vivo quantitative characterization panels (oscillatory rheology, rotational tribology, and texture analysis with bovine cartilage) were used for hydrogel product functional benchmarking, using equine synovial fluid as a biological control. Specifically, the retained experimental methodology enabled the authors to robustly assess and discuss various functional enhancement options for hyaluronan-based hydrogels (chemical cross-linking and addition of antioxidant stabilizing agents). The results showed that the Innoryos product, a niacinamide-augmented linear hyaluronan-based hydrogel, presented the best overall functional behavior in the retained experimental settings (high adhesivity and lubricity and substantial resistance to oxidative degradation). The Ostenil product was conversely shown to present less desirable functional properties for viscosupplementation compared to the other investigated products. Generally, this study confirmed the high importance of formulation development and control methodology optimization, aiming for the enhancement of novel OA-targeting product critical functional attributes and the probability of their clinical success. Overall, this work confirmed the tangible need for a comprehensive approach to hyaluronan-based viscosupplementation product functional benchmarking (product development and product selection by orthopedists) to maximize the chances of effective clinical OA management.

## 1. Introduction

Osteoarthritis (OA) is a highly prevalent pathology, marked by progressive articular cartilage structural and functional deterioration, as well as osteophyte formation, subchondral bone remodeling, and synovial membrane chronic inflammation [1,2]. It is noteworthy that knee OA eventually disrupts the entire joint system, progressively acting through an insidious and multifactorial pathophysiological cascade [1,2,3,4,5]. While many drivers and mechanisms of OA are currently under scientific investigation, compositional and functional analyses of healthy and diseased joints have guided the development of modern therapeutic solutions (i.e., without yielding a cure so far) [1,3]. Specifically, it is known that healthy cartilage tissue facilitates smooth bone movement by providing a low-friction, highly wear-resistant surface, and by acting as a shock absorber (i.e., with distribution of the forces applied to the joint during physical activities) [4,6]. Parallelly, viscoelastic knee synovial fluid (SF) plays a critical role in joint functionality, notably due to the biophysical attributes of its major constituents (e.g., lubricin, hyaluronic acid [HA], and other glycosaminoglycans [GAGs]) [6,7].

Importantly, HA constitutes up to ten percent of total GAGs in chondral tissues and is known to play an essential role in joint lubrication as well as in local physiological homeostasis [7,8]. It is noteworthy that biological HA synthesis is primarily performed by three transmembrane glycosyltransferase isoenzymes (i.e., hyaluronan synthases, HAS) and its degradation (i.e., itself modulated by patient age) is mediated by specific catabolic enzymes (i.e., hyaluronidases, HYAL) and/or by reactive oxygen species (ROS) [9,10,11,12]. In healthy knee joints, lubricin and HA confer to the SF various exceptional lubricating and shock-absorbing properties, resulting in optimal protection of the articular cartilage structures from excessive mechanical constraints and thus from accelerated wear [6,13]. Furthermore, the physiological presence of 1–4 mg/mL HA in knee joint SF contributes to maintaining its appropriate viscosity attributes and ensures optimal joint function by providing additional cushioning [13,14,15].

It is noteworthy that the clinical onset of OA is accompanied by marked SF qualitative degeneration, with decreased contents and mean molecular weight (MW) of HA, which results overall in diminished viscoelastic properties [15]. Hallmark clinical manifestations of OA, such as joint pain, articulation stiffness, motion limitation, and regional inflammation, are typically attributed to causative structural and functional alterations in the joint system [3,4]. As knee OA progresses over time, these symptoms exacerbate, often leading to significant patient disability and an impaired life quality [3,7,16,17]. From a therapeutic standpoint, exogeneous HA-based SF viscosupplementation was designed to alleviate several OA symptoms and has been safely clinically applied at large scales [18,19,20,21,22]. Notwithstanding, the long-term effectiveness of this intervention remains contested, mainly due to reported variability in patient responses, which are potentially attributable to differences in patient characteristics, disease severity, product formulation, or injection techniques [23].

From a functional standpoint, a major limitation of current HA-based viscosupplementation therapies is the rapid clearance of the named disaccharide polymer from the joint [9,10,11,12]. Therein, in addition to specific enzymatic degradation, the HA backbone is exposed to hydroxyl radical attacks, resulting in glycosidic bond scission [9,10,12,24]. Furthermore, ROS can also parallelly induce chondrocyte apoptosis and can stimulate the production and activation of the catabolic MMP-1 enzyme, thereby further exacerbating OA symptoms [25]. Within these pathophysiological constraints, and with the objective of avoiding dense injection regimens, developmental efforts have been focused on improving the local residence times of exogeneous HA and its global resistance to degradation [11,12,26,27]. Therefore, chemical derivatization strategies (e.g., backbone crosslinking, chemical functionalization) have been investigated to improve HA-based polymer stability [11,28,29]. Alternatively, co-formulation of HA with an appropriate antioxidant compound has been shown to significantly and intrinsically improve the stability of various hydrogel systems [12,28,30]. In addition, the in situ ROS-scavenging properties of the same antioxidants potentially contribute to preserve chondrocyte viability and extracellular matrix integrity, to reduce inflammation, and to provide a more conducive environment for HA-based product function [25,30,31].

The objective of the present work was to functionally benchmark four commercial HA-based viscosupplement products, focusing on critical rheological, lubricative, adhesive, and stability attributes. Specific commercial product selection criteria were applied, aiming to systematically include representatives from the main types of HA-based hydrogels in clinical use for OA management (i.e., linear [Ostenil], crosslinked [Synvisc], and antioxidant-supplemented [Ostenil Plus, Innoryos] preparations). Namely, the study focused on commercial viscosupplementation products requiring multiple injections and possessing proven clinical track records. In vitro and ex vivo characterization panels enabled to comparatively quantitatively assess the performance of various specific formulation-based functional enhancement modalities. Generally, the retained experimental methodology and the presented results confirmed the need for a comprehensive approach to viscosupplementation product functional benchmarking, in order to identify optimal preparations to be clinically used for OA management.

## 2. Results and Discussion

### 2.1. Technical Benchmarking of Viscosupplementation Product Parameters and Specificities

The modern therapeutic landscape for knee OA management is populated by diverse HA-based intra-articular injectables, which aim to replace and/or restore, albeit temporarily, the natural SF lubricating and cushioning properties [3,16,18,19,20,21,22]. For the needs of the present study, four different commercially available and clinically implemented HA-based viscosupplementation products were retained (i.e., Ostenil, Ostenil Plus, Synvisc, and Innoryos) and compared. Prior to the experimental functional benchmarking of the retained CE-marked medical devices (MD), the systematic gathering of relevant ad hoc technical documentation enabled a broad fact-based comparison of the investigated hydrogels (Table 1).

Although highly similar in terms of clinical indication and basic ingredient composition (i.e., injection-grade HA-based hydrogels), the investigated products are characterized by distinctive formulation-related attributes (e.g., HA polymer concentration, HA polymer MW distribution, type of incorporated additives; Table 1). Notwithstanding the technical variability outlined by the presented comparative work, all of the studied product formulation technologies were confirmed to be of current relevance in OA viscosupplementation management and in the corresponding guidelines (Table 1) [16,20,22,34,35,36,37].

Firstly, both Ostenil and Ostenil Plus products share an analogous HA source, with an average MW of 1.6 MDa (i.e., Ostenil Plus presenting higher HA contents, Table 1). Additionally, the latter contains mannitol as a stabilizing agent, to counteract the detrimental actions of free radicals [12]. Therefore, the mentioned product specificities enable the clinician to leverage therapeutic adaptability, wherein Ostenil Plus may potentially be preferred in more advanced OA clinical cases (Table 1). Secondly, Synvisc is composed of a hyaluronan-based complex (i.e., bi-component and chemically crosslinked Hylan G-F 20) with a bimodal polymer MW distribution (Table 1) [38]. The related formulation technology was designed to leverage the combined benefits of incorporating several HA MW classes, along with the increased system stability conferred by chemical polymer crosslinking, notably [38]. Thirdly, the Innoryos product presents a relatively wide monomodal HA polymer MW distribution, coupled to an elevated HA concentration (Table 1). The Innoryos hydrogel composition is further enriched with 1.5% niacinamide (i.e., or nicotinamide), for stability enhancement purposes and potentiation of the principal effects of the hydrogel in heavily damaged and inflamed osteoarthritic joints [36,37].

As regards the clinical administration regimens, standard weekly viscosupplementation injection protocols exist, yet the total number of administrations (e.g., 1–5 intra-articular injections) varies between products (Table 1). This spectrum of potential clinical uses enables the attending clinicians to instore therapeutic adaptability, enabling to fine-tune and tailor the treatment strategy based on individualized OA patient dynamics [16,19,20,21,22,23]. It is noteworthy that the currently available commercial viscosupplementation products are often classified by the number of injections (i.e., single injection, three injections, or five injections) which are required. Importantly, both non-adherence to the applicable clinical guidelines and suboptimal OA patient follow-up, combined with the described heterogeneity in specified administration regimens, are substantial sources of clinical failure [19,20,22]. While most current clinical guidelines recommend on average three viscosupplement injections at one-week intervals, recently launched products target optimized single-injection regimens, by leveraging higher HA concentrations or effective polymer crosslinking processes [16,22,38].

Generally, clinical orthopedic applications comprising HA-based viscosupplementation treatments have rapidly expanded (i.e., global market valued at USD 4.4 billion in 2021). Parallelly, considerable efforts have been allocated toward product formulation innovation and technological optimization, aimed both at commercial differentiation and at the enhancement of clinical efficacy. Marketed products differ mainly in HA types, HA MW distribution, and HA contents, but also in their additives or administration protocols [18,20,23,38]. Notable modern examples of alternative HA-based orthopedic viscosupplementation products are as follows, for comparison with the data reported in Table 1:

- Durolane (Bioventus, Durham, NC, USA), with a specified polymer concentration of 20 mg/mL, which uses the NASHA technology, and which was approved by the FDA in 2017;
- Synolis VA 80/160 (Aptissen, Plan-Les-Ouates, Switzerland), with a specified concentration of 20 mg/mL of high MW HA and 40 mg/mL sorbitol, which received a CE-mark in 2019;
- Gel One (Zimmer Biomet, Warsaw, IN, USA), which is crosslinked and has a specified HA concentration of 10 mg/mL.

### 2.2. Ex Vivo Characterization Results of Hydrogel Rheological Behavior

**Figure 1:**
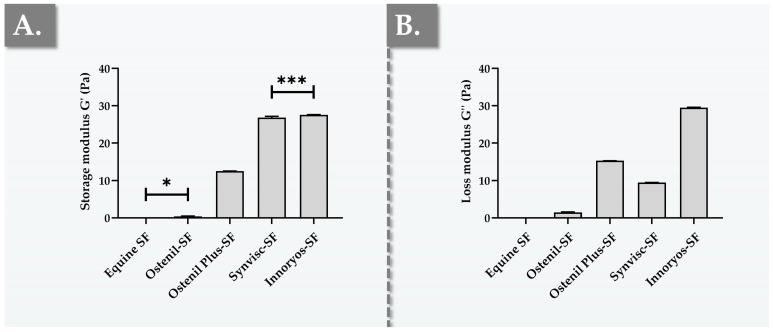
Results of ex vivo oscillatory rheology measurements for the four considered commercial hydrogel preparations. The samples were prepared by combination of hydrogel with equal volumes of fresh equine SF and were analyzed at 22 °C with a frequency of 0.5 Hz, simulating a normal walking condition. (**A**) Comparative quantitative determination of the G′ storage moduli. (**B**) Comparative quantitative determination of the G″ loss moduli. Data expressed as the η* complex viscosity are presented in Figure S1. The experimental tan δ values were of 1.5766 for equine SF, of 3.4438 for Ostenil-SF, of 1.2165 for Ostenil Plus-SF, of 0.3325 for Synvisc-SF, and of 1.0719 for Innoryos-SF. Measurements were performed in triplicate and standard deviations were reported as error bars around mean values. Statistically significant differences (“*” or *p*-value < 0.05) and highly significant differences (“***” or 0.0001 < *p*-value < 0.001) were found between the groups. Non-annotated inter-group differences were all found to be extremely significant (*p*-value < 0.0001). Detailed results of the statistical analysis are presented in Table S1. Pa, Pascals; SF, synovial fluid.

Basic functional characterization panels and the related quantitative manufacturing in-process controls for HA-based injectable hydrogels (e.g., dermal fillers, viscosupplements) heavily rely on various types of rheology [10,11]. Although being well adapted for applying quantitative analytical methods in defined and controlled hydrogel systems, in vitro rheology does not take into account the various biological parameters and components present in the knee joint [39,40]. In order to experimentally characterize the rheological behavior of the considered commercial preparations with enhanced biological relevance, the hydrogel samples were combined with fresh equine knee SF in order to obtain an ex vivo setup (i.e., mimicking the composition of the complex mixture present in the joint following viscosupplementation) [40]. Comparative determination of the main viscoelastic attributes of the obtained samples revealed important differences between the experimental groups for storage moduli and loss moduli (Figure 1).

Importantly, it is well known that in SF samples from osteoarthritic patients, the concentration and MW of HA is moderately to extensively decreased [15]. Therefore, direct injection of exogeneous HA into the knee joint capsule during orthopedic viscosupplementation aims notably to optimally replace or restore the rheological properties of healthy SF [7,20]. Thus, the rheological properties of the resulting exogeneous HA/endogenous SF combination (i.e., post injection) are primarily dependent upon those of the exogeneous hydrogel, as simulated in the ex vivo rheology model (Figure 1). While the viscoelastic attributes and the general rheological behavior of the viscosupplementation product itself (i.e., in the syringe) constitute critical quality attributes (i.e., predictive of in vivo functionality to some extent), it is important to bear in mind that such attributes may be drastically impacted by in situ mixing and diluting in patient SF [23,39,40].

The primary rheological parameters which are generally considered for the analysis of viscosupplementation products are the storage modulus (G′) and the loss modulus (G″) [11,12]. The storage modulus, or elastic modulus, represents an indication of the elastic or solid-like behavior of the material, while the loss modulus, or viscous modulus, indicates its viscous or liquid-like behavior [28]. Furthermore, the experimental use of an appropriate frequency in oscillatory rheology (e.g., 0.5 Hz for a walking knee; 2.5 Hz for a running knee) is essential for the methodological soundness and the biological relevance of the obtained quantitative data [28]. By extension, any means to further approximate the in vivo situation (e.g., product combination with ex vivo SF) may additionally be considered as an enhancement to the biological relevance of the experiments and may contribute to limit animal experimentation (Figure 1).

In the retained experimental rheological setup, the undiluted equine SF presented relatively low values for both moduli (i.e., 0.025 Pa for G′ and 0.039 Pa for G″), reflecting its predominantly liquid-like behavior (Figure 1). The product closest to the SF control (i.e., rheology-wise) was found to be Ostenil, which demonstrated the lowest values for both moduli (i.e., 0.43 Pa for G′ and 1.49 Pa for G″) among the four investigated products (Figure 1). In comparison, the obtained G′ and G″ values for Ostenil Plus were approximately 29 times and 10 times higher than those of Ostenil, respectively (Figure 1). These significant differences may be predominantly attributed to the differential HA concentration, which is effectively doubled in Ostenil Plus as compared to Ostenil (Table 1). Overall, the obtained rheology results were found to be significantly lower in average value as compared to the available literature sources, where the differences may be partly attributed to the choice of the analytical method and mostly to the sample dilution with the equine SF, as expected [41,42,43].

As concerns Synvisc, the results showed that G′ values were twice those of Ostenil Plus (Figure 1). However, it is interesting that the G″ values of Synvisc were found to be 1.5 times lower than those of Ostenil Plus (Figure 1). Specifically, the cross-linked hydrogel nature of Synvisc was clearly confirmed by its rheological behavior, with a storage modulus nearly three times greater in value than its loss modulus (i.e., 26.81 Pa for G′ and 9.46 Pa for G″, Figure 1) [41]. Notably, the presented rheological characterization results of Synvisc were generally congruent with those of other research groups [44,45]. Importantly, the experimental results reported for Synvisc mixed with equine SF were in concordance with the previous specific literature reports, wherein the same product was mixed with human SF in equal proportion [40].

Finally, Innoryos exhibited G′ values in the same range as Synvisc, with statistically significantly higher G′ values (i.e., +3%) in the retained experimental setup (i.e., 27.54 Pa vs. 26.81 Pa, Figure 1A). Furthermore, Innoryos displayed G″ values three times higher than those of Synvisc, two times higher than those of Ostenil Plus, and approximately 20 times higher than those of Ostenil (Figure 1B). Interestingly, the relevant literature sources report that, at a frequency of 2.5 Hz, the values of normal human SF may reach 19 Pa for G′ and 10 Pa for G″ [40]. Therefore, based on the viscoelastic behaviors of healthy human SF, it is possible to set forth that Synvisc and Innoryos are the most adequate viscosupplementation products (i.e., among those investigated herein) from a rheological viewpoint (Figure 1). It is of further note that several of the literature sources have reported experimental tan δ (i.e., G″/G′ ratio) values slightly below 1.0 for healthy human SF [40]. In this regard, Synvisc was experimentally found to behave as a gel-like material, with tan δ values close to the available reference values for healthy human SF (Figure 1) [40].

Along with the evolving scientific understanding of rheology, its use as an analytical and functional tool has continuously accompanied the developmental efforts aiming to innovate and to further improve orthopedic viscosupplementation formulations [28]. Notably, many product formulations have been designed to incorporate additives, cross-linking processes, or chemical modifications of the HA backbone aiming to enhance their rheological behavior, eventually aiming for clinical outcome enhancement and the procurement of longer-lasting relief [28,29]. Importantly, recent systematic reviews and meta-analyses have shown that higher viscoelastic properties generally correlate with longer HA-based injectable product retention times within the joint space, providing prolonged OA symptomatic relief. Conversely, lower viscoelastic properties generally imply the need for more frequent intra-articular injections in order to guarantee similar levels of clinical efficacy [46,47,48].

### 2.3. Ex Vivo Characterization Results of Hydrogel Lubrication Capacity

**Figure 2:**
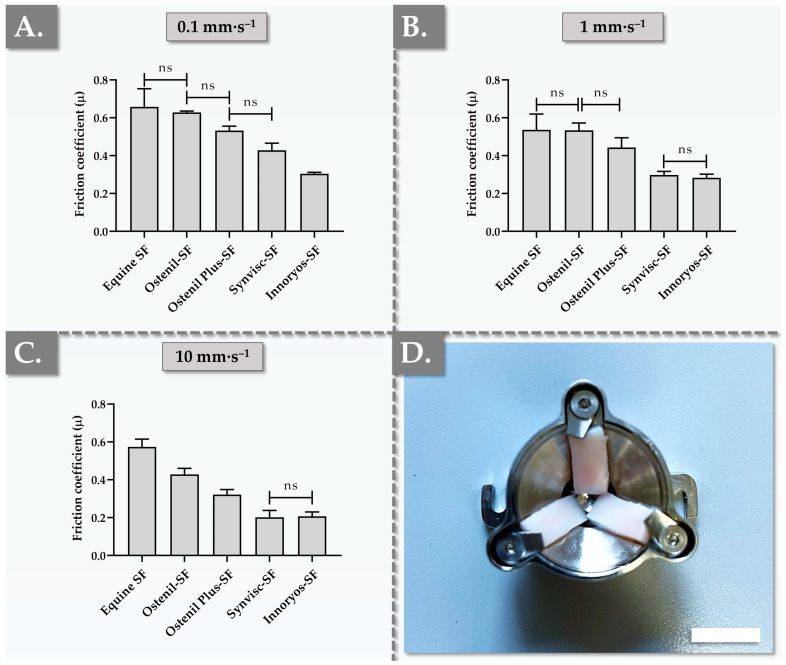
Results of ex vivo rotational tribology measurements for the four considered commercial hydrogel preparations. The samples were prepared by combination of hydrogel with equal volumes of fresh equine SF and were analyzed at 37 °C using various sliding velocities. Comparative quantitative determination of µ friction coefficients within the system under rotation at 0.1 mm·s^−1^ (**A**), at 1 mm·s^−1^ (**B**), and at 10 mm·s^−1^ (**C**). (**D**) Ex vivo rotational tribology setup, showing the lower portion of the tribology cell equipped with three samples of fresh load-bearing bovine articular cartilage. Scale bar = 10 mm. Measurements were performed in triplicate and standard deviations were reported as error bars around mean values. Statistically non-significant differences (“ns” or *p*-value > 0.05) were highlighted as appropriate between the experimental groups. Non-annotated inter-group differences were all found to be very significant (*p*-value < 0.01). Detailed results of the statistical analysis are presented in Table S2. ns, non-significant; SF, synovial fluid.

As one of the major physiological functions of SF is to reduce inter-surface interactions in the joint via the enhancement of local gliding properties, the intrinsic lubrication capacity of a viscosupplementation product constitutes a critical functional attribute [5,6,7,8,13,14]. As previously exposed in the ex vivo rheological experiments of the study, the in situ combination of the exogeneous therapeutic hydrogel with the locally present SF may substantially affect most (if not all) functional parameters of the system, as compared to a controlled in vitro system [40,45]. Therefore, the combination of the hydrogel products of interest with equine SF was performed again in the tribological setup, in order to approach the composition of viscosupplemented SF, before performing the measurements. Furthermore, in order to bring the experimental setup as close as possible to an in vivo situation (i.e., from a structural and chemical composition standpoint), the retained tribology cell was crafted to contain bovine load-bearing articular cartilage surfaces. The obtained experimental results confirmed that, compared to undiluted SF and in all experimental setups, the viscosupplemented sample groups were characterized by tendential or significant reductions in µ friction coefficients (Figure 2).

From a mechanical viewpoint (i.e., one of the mechanical facets of joint function), exogeneous viscosupplements act as lubricants and shock absorbers, principally countering the frictional forces generated during joint movement related to dynamic activities [13,14,15]. As elevated friction may be translated into perceived discomfort or pain, the lubrication capabilities of the injected hydrogel may significantly determine its eventual success in mitigating OA symptoms [15]. Experimentally, the µ friction coefficient of equine SF decreased as the set sliding velocity increased, as expected (Figure 2). At all of the investigated sliding velocities, the Ostenil-SF mixture presented the highest coefficients of friction, thereby displaying the lowest lubrication capacities among the four benchmarked hydrogel products (Figure 2). Generally, the µ coefficient of friction of each commercial hydrogel product decreased along with the increase in set sliding velocity, as expected (Figure 2). Despite the presence of a trend toward µ reduction in the Ostenil Plus group compared to the Ostenil group at sliding velocities of 0.1 mm·s^−1^ and 1 mm·s^−1^, no significant differences were found. However, at a sliding velocity of 10 mm·s^−1^, the lubrication capacity of Ostenil Plus was found to be significantly superior over that of Ostenil (i.e., coefficient of friction values of 0.32 and 0.43, respectively, Figure 2). This behavior could potentially and partly be explained by the difference in HA contents between the two products, as the two-fold higher concentration of HA in Ostenil Plus confers relatively high viscosity values (Figure 1, Table 1).

In the retained ex vivo tribology setup, Synvisc exhibited superior lubrication capacities as compared to the two types of Ostenil products, with significantly lower µ values at all sliding velocities in comparison with Ostenil and at 1 mm·s^−1^ and 10 mm·s^−1^ in comparison with Ostenil Plus (Figure 2). Interestingly, the relevant literature reports higher lubrication capacity values for products such as Synvisc and Hymovis (i.e., which contains HYADD4 at a concentration of 8 mg/mL, Fidia Farmaceutici, Abano Terme, Italy) at sliding velocities of 0.1 mm·s^−1^, 1 mm·s^−1^, and 10 mm·s^−1^, compared to linear HA-based products like Euflexxa (Ferring Pharmaceuticals, Saint-Prex, Switzerland) [49].

As concerns the Innoryos group, the investigated samples displayed significantly lower overall values at the lowest sliding velocity, with µ values 2 times lower than those of Ostenil, 1.8 times lower than those of Ostenil Plus, and 1.4 times lower than those of Synvisc (Figure 2A, Table S2). Interestingly, at sliding velocities of 0.1 mm·s^−1^ and 1 mm·s^−1^, the recorded relative reductions in µ values were less pronounced in the Innoryos group as compared to the other viscosupplements (i.e., coefficients of friction of 0.30 at 0.1 mm·s^−1^ and 0.28 at 1 mm·s^−1^, Figure 2). Generally, at sliding velocities of 1 mm·s^−1^ and 10 mm·s^−1^, Innoryos demonstrated significantly stronger lubrication abilities than those of the two types of Ostenil products, yet the lubrication performance of Innoryos was found to be similar to that of Synvisc (Figure 2).

Importantly, the available literature reports showed no clear dependency between the HA polymer MW and the levels of measured friction in the cartilage-on-glass contact but highlighted the importance of the interaction between the considered HA and other SF constituents [50]. Such pre-existing elements contributed to supporting the retained ex vivo methodology for obtaining the presented tribological datasets (i.e., use of equine SF and bovine cartilage). Nevertheless, in general, the viscoelastic properties of a hydrogel system play a crucial role in the lubrication abilities of the same system, in addition to specific mechanisms (e.g., hydration lubrication of articular cartilage surfaces) [51,52]. As presented hereabove, Ostenil demonstrated the highest coefficient of friction values among the four products, followed by Ostenil Plus, Synvisc, and Innoryos (Figure 2). As the sliding velocity increased, and while the overall ranking remained consistent, Synvisc and Innoryos exhibited similar µ values, despite Innoryos having overall higher viscoelastic properties (Figure 1 and Figure 2).

In order to augment the robustness of the presented experimental tribological datasets and in the context of the product functional benchmarking assays in particular, the rotational tribology experiments were performed again using an in vitro setup. In detail, the tribological cell was equipped with PDMS plates (i.e., widely used material in OA modeling) instead of the articular cartilage plates and the samples were composed of undiluted commercial hydrogel products [53,54]. The obtained results were similar in trend and in absolute values between the ex vivo and the in vitro setups, with slightly lower coefficients of friction recorded in the in vitro experiments (Figure S2). The overall similar behavior of the samples in the two different tribology systems was interpreted positively from a methodological standpoint, yet the respective differences in quantitative results enabled to illustrate the importance of setup selection for optimal relevance of the produced datasets (Figure 2 and Figure S2). Specifically, it was assessed that the standardized in vitro setup could potentially be further used for large-scale screening purposes, while the adapted ex vivo setup may be preferred for in-depth product functional qualification with enhanced translational relevance. In detail, it is well known that molecular interactions between HA-based therapeutic hydrogels and biological constituents of the joint play key roles in overall system lubrication, confirming the tangible interest of using ex vivo analytical workflows for in-depth product assessments [55,56,57]. It is noteworthy that due to the highly specific nature of the retained ex vivo tribology setup, no direct or quantitative comparison with the available literature reports was performed for the presented coefficient of friction datasets [57].

In order to further explore the potential interdependency between the viscoelasticity and lubricity attributes of the investigated experimental samples, multiparametric analyses of correlation were performed. Specifically, despite the complexity of the lubrication phenomenon, the η* complex viscosity, the G′ elastic modulus, and the G″ viscous modulus were individually correlated with the experimental µ coefficients of friction at each sliding velocity (Figure S3). Therein, the obtained values for the R^2^ coefficients of correlation were found to be generally higher for the elastic modulus and the complex viscosity parameters, as compared to the viscous modulus parameter (Figure S3). Furthermore, it is noteworthy that as the sliding velocity increased, the level of correlation between the friction coefficients and the viscoelastic parameters diminished (Figure S3).

Interestingly, the lubricating capacities of injectable viscosupplementation products are specifically considered to be more predictive of clinical outcomes than their viscoelastic properties [49]. This element underscores the importance of developing and using viscosupplements characterized by optimal lubrication abilities, in order to maximize the potential for therapeutic success [57]. In detail, while the maintenance of appropriate viscoelasticity attributes is undeniably a critical functional factor for viscosupplements, ensuring effective lubrication of the treated joint is of paramount importance [7,19]. Indeed, enhanced lubrication capabilities can potentially mitigate/delay articular surface wear, reduce pain, and improve joint mobility. Therefore, as the field of viscosupplementation continues to evolve, the focus on optimizing product lubrication properties remains at the forefront, with the hope of translating enhanced hydrogel functionality into bettered clinical outcomes [49,53].

### 2.4. Ex Vivo Characterization Results of Hydrogel Bio-Adhesion Capacity

In addition to the previously discussed critical functional attributes (i.e., viscoelasticity, lubricity) of HA-based OA-targeting viscosupplements, considering the product bio-adhesivity parameter is of prime importance, especially in moderate to advanced OA [58,59]. This concept is best explained by the fact that even the ideal lubricant would perform poorly overall if its residence time is short and its clearance is rapid. Such considerations are especially impactful in progressive degenerative affections such as knee OA, wherein the duration of the therapeutic effect of the intervention represents a major factor for clinical success [28]. Notwithstanding the fact that HA-based hydrogel clearance from the joint is mediated by several mechanisms and factors such as system degradation or cohesivity, physical residence at the administration site may be enhanced by optimizing general product bio-adhesivity attributes [9,23].

**Figure 3:**
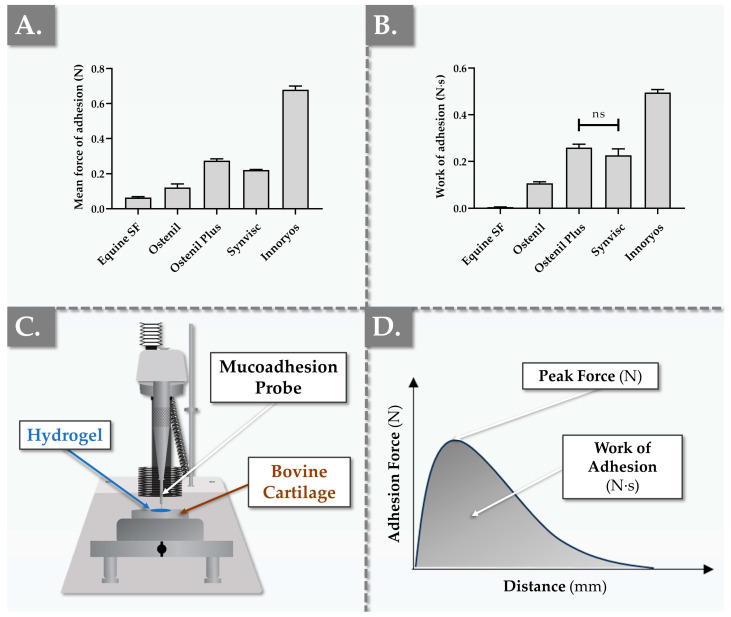
Results of ex vivo bio-adhesion measurements for the four considered commercial hydrogel preparations on bovine tibial articular cartilage. The undiluted samples were analyzed at 37 °C with a constant detachment speed of 2 mm·s^−1^ between a steel mucoadhesion probe and a fresh plane portion of load-bearing cartilage. (**A**) Comparative quantitative determination of the peak force of adhesion of the samples. (**B**) Comparative quantitative determination of the work of adhesion of the samples. (**C**) Schematic and annotated representation of the experimental ex vivo bio-adhesion setup. (**D**) Schematic and annotated representation of the obtained bio-adhesion data. Measurements were performed in triplicate and standard deviations were reported as error bars around mean values. Statistically non-significant differences (“ns” or *p*-value > 0.05) were highlighted as appropriate between the experimental groups. Non-annotated inter-group differences were all found to be very significant (*p*-value < 0.01). Detailed results of the statistical analysis are presented in Table S4. N, Newtons; N·s, Newton seconds; ns, non-significant; SF, synovial fluid.

In order to further functionally benchmark the commercial hydrogel products of interest, an adapted ex vivo mucoadhesion setup was used to comparatively assess the bio-adhesion performance of the samples via a tack test. Experimental results were gathered using two types of bovine articular cartilage (i.e., tibial and meniscal surfaces, Figure 3 and Figure S4).

It is noteworthy that despite the presence of quantitative differences in the obtained bio-adhesion values between the two retained ex vivo setups (i.e., tibial versus meniscal cartilage), the overall comparative performance profile of the investigated products was conserved (Figure 3 and Figure S4). In the tibial articular cartilage setup, the equine SF control group exhibited an average force of adhesion (i.e., or “stickiness”) and a mean work of adhesion (i.e., or “cohesivity”) of approximately 0.063 N and 0.005 N·s, respectively (Figure 3). All four undiluted commercial hydrogel products displayed higher forces of adhesion compared to pure equine SF in the same setup, as expected (Figure 3A). Specifically, Ostenil exhibited the lowest values for both the mean force of adhesion and the mean work of adhesion. These results were found to be in line with the relative performance of Ostenil in terms of viscoelastic properties and lubricative capacity (Figure 1 and Figure 2). In direct comparison, Ostenil Plus displayed more than double the adhesion force and work of adhesion over Ostenil, suggesting that the increased HA content in Ostenil Plus could potentially favor increased product retention and residence time in the treated joint (Figure 3).

Synvisc, which presented an average adhesion force of 0.221 N and a mean work of adhesion of 0.227 N·s, was found to occupy an intermediate position among the four investigated commercial products, from a bio-adhesion viewpoint (Figure 3 and Figure S4). However, the Innoryos product was found to distinctly stand out in terms of bio-adhesion, as it displayed the highest mean force of adhesion and work of adhesion values among the tested samples (Figure 3 and Figure S4). Such results suggested that Innoryos is characterized by a particularly robust affinity for joint chondral surfaces. By extension, this inherent bio-adhesive attribute was interpreted positively, as it confers the potential to deploy extended therapeutic benefits within the intra-articular environment [59,60].

From a methodology standpoint, the force of adhesion and work of adhesion values of the equine SF (i.e., internal control) were used as references in the retained experimental ex vivo bio-adhesion setup (Figure 3). This control group was included and was favored over the use of a source from the literature for theoretical reference values, as the experimental setup did not correspond to readily available reports. The experimental bio-adhesion data obtained for the various HA-based product groups were interpreted positively in light of the recorded comparative product performance panel (Figure 3 and Figure S4). Therein, enhanced product bio-adhesivity on cartilage tissues (i.e., compared to SF) potentially augments the residence time of the hydrogel within the joint, thereby extending the duration of the local therapeutic effects by slowing down hydrogel clearance. Furthermore, the intergroup differences could be explained from a mechanistic viewpoint (i.e., structure–function relationship), where the various means of hydrogel functional enhancement (e.g., cross-linking, antioxidant addition) could procure enhanced adhesivity attributes (Figure 3). Nevertheless, the adhesive performance results of the investigated hydrogel systems were mainly linked to the physical properties of the gel, including the complex viscosity (Figure S1) and the HA polymer concentration (Table 1). Overall, the experimental values obtained in the retained ex vivo setup confirmed the desirable adhesive attributes of the investigated HA-based products, which may be set forth to support the efficacy of the same products in OA applications [27,60].

Generally, an HA-based hydrogel characterized by high viscosity values can be expected to be highly adhesive. However, when assessing highly deformable semisolids, it should be underlined that this bio-adhesion practically encompasses not only the pure interfacial adhesion force, but also the force required to deform the hydrogel until the detachment point is reached, the second being related to viscous and elastic gel components. A potential mechanism underlying the interfacial part of this phenomenon is governed by molecular interactions. In detail, as HA is a high-MW polymer, its macromolecules have the potential to establish multiple types of chemical interactions with cartilage surface proteins, thereby promoting its adhesion [60]. From a developmental viewpoint, and although several chemical modifications exist to enhance HA adhesive properties, a notable example is the grafting of catechol functional groups onto the HA backbone. In this context, the available literature reports notably suggest that hydrogels formulated with linear HA tend to demonstrate good mucoadhesion but weak adhesion to cartilage tissues [60,61].

With regard to detailed internal modes of actions, the role of niacinamide, as present in the Innoryos product and conferring significant adhesive properties, is not yet entirely elucidated (Figure 3, Table 1). It is postulated specifically that niacinamide could play a role in molecular interactions with cartilage, such as through hydrogen bonding.

While adhesive hydrogels have been largely and commonly evaluated for use in combination products, specifically those designed to optimally deliver drugs, adhesivity plays a multifactorial critical role for intra-articular viscosupplementation [29,60]. Specifically, good adhesivity ensures that the injected hydrogel remains within the joint space longer, providing extended therapeutic effects and thereby potentially reducing the reinjection frequency. Mechanistically, adherent HA-based systems provide a stable, lubricating layer, thus reducing friction between opposing cartilage surfaces. Furthermore, the adherent HA layer might act as a mechanical and biochemical protective shield, offering further cartilage tissue protection and potentially decelerating OA progression [49,60,61,62,63,64]. Such fundamental elements are especially important for viscosupplementation applications, as cartilage surfaces are never perfectly smooth and often present a certain degree of roughness, especially in patients suffering from OA [65,66]. Therein, the artificial presence of an exogeneous adhesive hydrogel could aid in filling such structural irregularities or in promoting aggregation. Consequently, this could enable the hydrogel to establish a boundary layer on the treated cartilage surface, potentially enhancing the therapeutic effects of the treatment [62,63,65]. Overall, advanced knowledge of the adhesive properties of HA-based systems is deemed to be essential not only for applications in drug delivery, but also for optimization of their therapeutic role in joint lubrication and protection/repair [55,58,59,60,61].

### 2.5. In Vitro Results of Hydrogel Resistance to Oxidative Degradation

As previously reported in the bio-adhesivity section of the study, major drivers of viscosupplementation product clinical efficacy reduction consist in factors which lower the in situ product residence time, as well as any catabolic processes which negatively impact the critical functional attributes of the system [11,12]. Although the specific enzymatic catabolic processes of HA-based system clearance (e.g., as mediated by HYAL enzymes) are instrumental in the progressive loss of efficacy of the administered product, concomitant oxidative processes of degradation may be interpreted at least as equally important factors [67]. This is especially verified in cases where the local inflammatory component is elevated, thereby precipitating the action of ROS-mediated hydrogel damage. Furthermore, HA-based system oxidative degradation may generally occur after product conditioning but before clinical administration (i.e., for a number of stability-related reasons), confirming the specific relevance of evaluating the resistance of the investigated commercial hydrogel products to strong oxidative stimuli [12,67]. Overall, the robust assessment of HA-based hydrogel system stability and general resilience in adverse conditions is of key design, manufacturing, and clinical interest [68,69].

**Figure 4:**
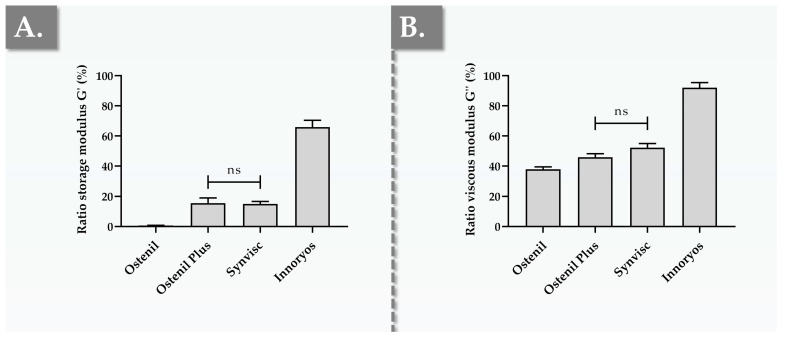
Results of accelerated stability studies expressed as endpoint residual fractions of rheological attributes for the four considered commercial hydrogel preparations exposed to a strong oxidant source. The samples were prepared by exposure of undiluted hydrogel to H_2_O_2_ for 30 min and were analyzed in oscillatory rheology at 37 °C with a frequency of 0.5 Hz. (**A**) Endpoint residual fractions of the G′ storage moduli. (**B**) Endpoint residual fractions of the G″ loss moduli. Data expressed as the residual fraction of “* complex viscosity values are presented in Figure S5. Measurements were performed in triplicate and standard deviations were reported as error bars around mean values. Statistically non-significant differences (“ns” or *p*-value > 0.05) were highlighted as appropriate between the experimental groups. Non-annotated inter-group differences were all found to be very significant (*p*-value < 0.01). Detailed results of the statistical analysis are presented in Table S6. min, minute; ns, non-significant.

The results of the in vitro oxidative challenge assays revealed that the four considered hydrogel systems behave quite differently in the presence of a strong and standardized oxidant source (i.e., 30% H_2_O_2_, Figure 4).

As the structural integrity of HA-based hydrogel systems is particularly affected by ROS such as H_2_O_2_ in inflammatory in vivo environments, this specific oxidant was retained for the presented stability experiments. At the end of the H_2_O_2_ challenge phase, Ostenil displayed a drastic reduction in G′, with post-exposure ratios dropping by more than 99% (Figure 4A). Although the corresponding relative reduction in G″ was recorded as more moderate (i.e., 62%), Ostenil displayed the overall weakest resistance to oxidative degradation (Figure 4B). Such results, which were congruent with the existing literature reports, underscored the vulnerability of Ostenil to oxidant-mediated catabolism, which may be logically explained by the relatively low HA content and the absence of protective polymer crosslinking measures or antioxidant excipients (Table 1) [28,70]. Specifically, HA-based polymer chemical grafting or the incorporation of complex antioxidant excipients were previously shown (i.e., in the same oxidative challenge setup) to significantly protect the rheological properties of the system [12,28,70].

In contrast to Ostenil, Ostenil Plus exhibited mean residual G′ ratios around 15.5%, suggesting a significantly enhanced resistance toward oxidative degradation, as expected (Figure 4A). Furthermore, the mean residual G″ ratios for Ostenil Plus were recorded at 48.8%, which was determined to be markedly better in terms of resistance compared to Ostenil (Figure 4B). Here again, the existing literature reports supported the presented findings, outlining the role of mannitol in the Ostenil Plus formulation as an effective antioxidant which shields HA from degradation [12,67].

Interestingly, despite being crosslinked (i.e., a feature expected to robustly enhance resistance toward oxidative degradation), Synvisc’s residual G′ and G″ ratios were comparable in value to those of Ostenil Plus (Figure 4). These results were interpreted as highlighting the tangible functional advantage of incorporating antioxidants, a simple and effective preemptive countermeasure to the inevitable action of oxidative degradation [67]. Furthermore, Innoryos demonstrated the most robust stability in the retained experimental setup, with G′ and G″ reductions of 44% and 8%, respectively (Figure 4). As HA contents are comparable between Ostenil Plus and Innoryos, the significant advantage of the latter in terms of resistance to oxidative breakdown may be attributed for the most part to the action of niacinamide (Figure 4, Table 1).

Niacinamide, also known as nicotinamide, is a potent, well-recognized antioxidant [33,70,71,72]. While its precise interactions with HA-based polymers warrant further research, the available literature often associates the action of niacinamide with significant antioxidant effects on skin cells, primarily by suppressing ROS generation and by lowering the NADP^+^/NADPH ratio [33,71,72,73]. Specifically, in an experimental determination of antioxidant activity using the DPPH (i.e., 2,2-diphényl 1-picrylhydrazyle) method, some authors have shown that niacinamide expressed antioxidant activity comparable to that of ascorbic acid [74]. However, the antioxidant activity of niacinamide alone could not be set forth herein as the main mechanistic explanation for the enhanced stability of Innoryos compared to Ostenil Plus (Figure 4). Specifically, while the experimental TEAC values of mannitol and niacinamide were found to be significantly different from that of the control group, no statistically significant difference in antioxidant activity was found between the two excipients (Figure S6). Such results should warrant the further use of orthogonal analytical methods for antioxidant activity determination, as well as specific investigation into the molecular interactions occurring between niacinamide and HA polymers.

It is noteworthy that the comparative investigation of antioxidant activities between different compound types is not straightforward and depends largely on the retained analytical method. Several commercial assays are currently available for antioxidant activity quantification (e.g., Trolox, DPPH, ferric reducing antioxidant power [FRAP], or cellular antioxidant activity assays) and these may be classified based on their respective mechanisms of function [75]. Furthermore, one must exercise caution when designing a new hydrogel formula incorporating an antioxidant excipient, as some molecules exhibit antioxidant properties, yet they do not protect HA from oxidative degradation. For instance, ascorbic acid (i.e., vitamin C) in solution at 1 mg/mL is known to be a potent antioxidant compound, yet such amounts degrade HA following incorporation in linear polymer-based hydrogels.

### 2.6. Commercial Viscosupplementation Product Functional Benchmark Summary and Overall Comparative Assessment

Aggregation of the experimental data presented herein (i.e., oscillatory rheology, rotational tribology, bio-adhesion, and oxidative degradation) clearly outlined, from a product-centered functional viewpoint, that the use of 1% linear HA without additives might qualify as suboptimal for orthopedic viscosupplementation (Figure 1, Figure 2, Figure 3 and Figure 4). Indeed, close comparative analysis of the collected data clearly indicated that the use of high HA concentrations, intermediate to high HA MW, and appropriate means of hydrogel system stabilization resulted in the tangible enhancement of functional performance (e.g., Ostenil Plus, Synvisc, and Innoryos versus Ostenil). A synoptic overview of the obtained original data enabled to identify Innoryos (i.e., among the four investigated hydrogel products, within the limits of the retained experimental setups) as the overall best-performing system from an in vitro and ex vivo functional viewpoint (Table 2).

The obtained results were furthermore found to be congruent with applicable principles of the existing body of knowledge around therapeutic HA-based hydrogels [12,18,60]. Specifically, it was confirmed that by elevating system viscosity values (i.e., either by increasing the HA concentration or through crosslinking), beneficial effects were procured in terms of functional performance (Table 1 and Table 2). Once injected, a viscosupplement will mix with the SF in situ. Therefore, it is crucial to examine its rheological properties when combined with SF. Depending on the product formulation, once the hydrogel is blended with the SF, specific properties can degrade at different rates and the system may therefore exhibit distinct behaviors and effects [40,42,45].

Furthermore, it was confirmed that the incorporation of an antioxidant excipient (e.g., a polyol in Ostenil Plus or a potent small molecule in Innoryos) procured significant functional benefits, especially regarding oxidative stress-related degradation (Figure 4) [12,67]. Generally, the selection of an antioxidant compound or antioxidant complex presenting additional and complementary properties may potentially further enhance the functional performance and the clinical effects of therapeutic HA-based hydrogels [76,77]. The selection of an appropriate additive can significantly influence the stability of a viscosupplement [67,77]. Therein, if the additive functions as an antioxidant, it becomes essential to discriminate between its antioxidant potency and its capacity to shield HA from the degradation caused by ROS. This differentiation is critical to validate the functional role of the studied excipient and to be able to set forth the prolonged effects of the viscosupplement.

Importantly, the experimental results reported in this study enabled to underscore a link between system chemical structural attributes and system functional attributes (i.e., from a viscosupplementation function standpoint). Specifically, the formulation-related rationale for the use of high HA polymer contents, HA polymer chemical cross-linking, or the incorporation of antioxidants was experimentally confirmed to procure significant functional and stability enhancements to the respective hydrogel systems (Figure 1, Figure 2, Figure 3 and Figure 4). In detail, the presence of high HA polymer quantities (e.g., in Ostenil Plus vs. Ostenil, Table 1) probably favors increased non-ionic interactions between HA chains themselves and between HA chains and the components of the studied interface, resulting in enhanced viscoelastic and lubricative properties. Then, the presence of chemical cross-linking (e.g., in Synvisc) inherently ensures strong interactions within the polymer network, providing a more defined system framework at the interface [78]. The cross-linking process enables to enhance mechanical properties, reduce degradation rates, and reduce swelling, which will have an impact on the resulting system adhesivity [79]. Finally, the use of small molecule antioxidants in relatively abundant amounts (e.g., niacinamide in Innoryos, Table 1) probably mediates enhanced non-ionic interactions within the HA polymer-based hydrogel system, favoring the adoption of an optimal HA chain supramolecular conformation and thereby procuring an enhanced functionality and strong resistance to degradation. However, further mechanistic studies are necessary for a strengthened comprehension of structure–function relationships. This fact is illustrated by the current state of specific research, where the effects of HA polymer MW on the interactions with cartilage surfaces or with SF are not yet fully understood [80].

In this study, four critical functional parameters were investigated using in vitro and ex vivo setups which were designed to closely mimic in vivo conditions. Additionally, alternative performance tests are available for injectable HA-based therapeutic hydrogels, as listed in Table S8. Notably, it is set forth that evaluating the injectability and swelling ratio of a viscosupplement is not critical from a functional or a technical standpoint. Indeed, orthopedic hydrogel-based applications generally require the use of wide-gauge needles (i.e., 18–25 G), which do not render most hydrogels difficult to inject. Regarding the swelling ratio, most viscosupplementation products on the market are based on linear HA, and this test may not be suitable for such products. However, when it comes to HA-based dermal fillers, these tests are of paramount importance in assessing their performance, due to the different administration modalities and the nature of the desired effects. Generally, it may be assessed as highly beneficial to conduct relevant performance tests using appropriate controls (i.e., commercial products) during the development of new HA-based viscosupplements (Table S8).

### 2.7. Study Limitations and Future Perspectives

The main identified limitations of the present study comprised the use of only four different commercial products and a limited number of functional characterization assays. The related choices were made in order to appropriately represent the main types of different HA-based hydrogels which are clinically used in OA (i.e., linear, crosslinked, antioxidant-supplemented), while focusing on the most relevant functional parameters (i.e., those generally thought to be predictive of clinical efficacy and of major regulatory importance, Table S8). Furthermore, no correlations were made with the available clinical efficacy results of the specified commercial products, to potentially establish a link between ex vivo performance and an enhanced probability of clinical success.

Overall, while significant differences were experimentally outlined herein, the effective clinical added value of the different product types must be confirmed at large scales, especially for more recent products. It is noteworthy that Ostenil was characterized as the overall worse-performing group in the retained comparative experimental setups, yet the 25 years it has been on the market and the many patients which were successfully treated with this preparation should also be taken into account in holistic analyses. Therefore, further enhancement of the scientific comprehension of OA-governing factors and functional mechanistic elements of OA-targeting products are still required. Specifically, the potential ability to predict clinical efficacy based on an appropriate product functional characterization panel is currently highly appealing, notably for late-stage product attrition rate mitigation and drastic rationalization of investigative animal experimentation.

From a methodological standpoint, the presented study was limited by the fact that no experimental characterization of the actual HA polymer MW distribution was performed for the retained viscosupplements. Specifically, the comparative benchmarking of general product parameters and characteristics was based on manufacturer-provided information (Table 1). Indeed, as the comparative rheological profiles of the four product groups behaved as expected based on the listed formulations of the products (Figure 1), no additional experimental characterization work concerning the MW distributions was performed. Such analyses could be further undertaken using size-exclusion chromatography-based workflows, which would be well adapted for linear HA-based polymers and would require some technical adaptation in the case of cross-linked HA polymer systems (e.g., Synvisc) [81].

Another limitation of the study consisted in the absence of experimental biological data (e.g., cell viability assay, use of OA biomarkers) to complement the ex vivo functional assay results. It is noteworthy that such data were not included within the scope of the presented work, based mainly on the specified objective of the study as regards systematic and comprehensive functional investigation of the retained commercial products. Specifically, the considered HA-based hydrogels were all registered and commercialized as medical devices, which principally act via physical/mechanical mechanisms of action (e.g., reduction of friction, cushioning). While some ancillary biological mechanisms of action of the devices are not excluded, they do not constitute the necessary and sufficient components of product function/efficacy [28,77]. It is noteworthy that cell viability assays are part of product safety-related characterization within the corresponding development processes, yet this aspect was assessed to be of ancillary importance to the scope of the functional-oriented characterization of the presented study [28]. Furthermore, the designed product selection workflows within the study have limited the experimental focus to HA-based viscosupplementation products with a commercial and clinical track record, which implicitly attests to the appropriate validation of their safety profile during development phases. Furthermore, the prospective in vivo study of OA-related biomarkers and the comparative assessment of the performance of the different studied products in that regard would be of highest interest, yet the level of clinical resources necessary for such studies has set them outside of current local possibilities.

Finally, future perspectives opened by the present study comprise the further development and standardization of assays and methods to be used for the functional characterization of alternative hyaluronan-based hydrogel systems. Specifically, as a large proportion of HA-containing commercial products are destined for dermatological applications, a robust approach of relevant functional parameters within such applications (e.g., hydrogel system cohesivity and local resistance to physiological clearance) is of great interest [82]. Therefore, several in vitro and ex vivo experimental setups and methods may be devised, building on the methodology reported herein. Furthermore, as diverse antioxidant compounds are also included in many novel products such as mesotherapy-based dermal fillers, specific and standardized functional assessments of commonly used excipients (i.e., using ABTS decolorization assays and antioxidant assays with orthogonal methods) may shed some light on their effective added value, from formulation rationale and mechanistic viewpoints [83,84,85].

## 3. Conclusions

The therapeutic efficacy of HA-based viscosupplements in clinical OA management is intrinsically linked to their critical viscoelasticity, lubricative, and adhesive attributes, as well as their resilience against degradation. A major methodological advantage of the present study resided in the selection of critically relevant functional assay types for commercial product benchmarking, as well as the use of ex vivo setups wherever possible. Despite the limited number of analyzed commercial products, the main types of different HA-based viscosupplementation technologies were represented herein.

Among the investigated commercial preparations, Innoryos presented significantly superior bio-adhesive properties and demonstrated remarkable resistance toward oxidative degradation. The obtained datasets were interpreted to suggest, from a functional viewpoint, that Innoryos presented a potential for enhanced intra-articular therapeutic effect exertion. Therein, the contributory role of niacinamide was critically appraised, yet full mechanistic explanation of its mode of action was not possible.

Future prospects to the reported work comprise the systematic functional characterization of HA-based commercial hydrogels for cutaneous administration, which represent an important category of clinically administered products. Therefore, the comprehensive methodological approach adopted in this work, coupled to specific functional investigation of common hyaluronan hydrogel additives (e.g., antioxidants, viscomodulators), may contribute to orient product formulation development and preclinical evidence-based product selection by clinicians.

Overall, the obtained data confirmed and underscored the critical role of optimal hydrogel viscosity tuning and strategic incorporation of functional additives. Furthermore, strong focus was set on the methodological elements of commercial product benchmarking. Key consideration points were provided for function-oriented novel OA-targeting product development or for the selection of marketed injectable viscosupplementation products for implementation in orthopedic clinical practice.

## 4. Materials and Methods

### 4.1. Materials and Consumables Used for the Study

The materials and consumables which were used in the study were as follows: purified water and PBS buffer (Bichsel, Unterseen, Switzerland); Ostenil and Ostenil Plus (TRB Chemedica, Geneva, Switzerland; Lot TC0910ACA and Lot VK1002ALA, respectively); Synvisc (Sanofi Genzyme, Cambridge, MA, USA; Lot CRSP009Z); Innoryos (Albomed, Schwarzenbruck, Germany; Lot TA002F-01); Total Antioxidant Capacity (TEAC) Assay Kits, niacinamide, mannitol, and hydrogen peroxide 30% *w*/*w* (Sigma-Aldrich, Buchs, Switzerland); equine synovial fluid (i.e., aspirated from six fetlock joints, obtained after slaughter from healthy adult horses; Communal slaughterhouse, Delémont, Switzerland); and bovine cartilage tissues (Boucherie Chevaline du Vieux Carouge, Geneva, Switzerland).

### 4.2. Ex Vivo Rheological Behavior Setup

In order to mimic the in vivo composition of viscosupplemented SF for the assay, the considered commercial hydrogel products were combined in equal proportion to freshly harvested equine SF. The rheological behaviors of the obtained samples were determined in oscillatory rheology using a HAAKE Mars Rheometer (Thermo Fisher Scientific, Waltham, MA, USA) equipped with a Peltier cone-plate characterized by a C35 2°/Ti measuring geometry. All measurements were performed on volumes of 420 µL for the ex vivo samples and for the control groups (i.e., undiluted fresh equine SF). Measurements were performed at 22 °C with a constant oscillatory frequency of 0.5 Hz, simulating walking conditions [28]. Shear stress was set to 3 N/m^2^ in all the assays, to remain in the linear viscoelastic region (LVE). The experimental storage moduli (G′) and loss moduli (G″) were determined, using three experimental replicates for all the assays. A sample hood was used during the measurements, to minimize sample evaporation.

### 4.3. Ex Vivo Rotational Tribology Setup

In order to mimic the in vivo composition of viscosupplemented SF for the assay, the considered commercial hydrogel products were combined in equal proportion to freshly harvested equine SF. The lubrication capacities of the obtained samples were determined in rotational tribology using an MCR 302 rotational rheometer (Anton Paar, Graz, Austria) equipped with a T-PTD 200 ball-on-three-plates tribology cell. The instrument was equipped with a SoLi glass ball of 12.7 mm in diameter. The tribology cell was equipped with three plates made from the load-bearing surface of bovine articular cartilage, crafted to replace the original steel or polydimethylsiloxane (PDMS) plates of the manufacturer. All measurements were performed on volumes of 700 µL for the ex vivo samples and for the control groups (i.e., undiluted fresh equine SF). Measurements were performed at 37 °C using a normal force of 3 N, resulting in a maximum contact pressure of 290 kPa. The friction factor/coefficient μ, representing the interaction between both sliding surfaces, was measured at 0.1, 1, and 10 mm·s^−1^ sliding velocities. The entire assay was eventually repeated using PDMS plates instead of the bovine articular cartilage plates, using undiluted commercial products.

### 4.4. Ex Vivo Bio-Adhesivity Evaluation Setup

The bio-adhesivity attributes of the considered commercial hydrogel preparations were determined using an ex vivo texture analysis (tack test) setup. The presented bio-adhesion evaluation setup was previously reported using ex vivo skin samples (i.e., dermatological hydrogel characterization), yet the introduction of load-bearing cartilage tissue in the design corresponded to an original workflow [82]. Plane bovine adult cartilage tissue samples were retrieved from the meniscus and tibial portions of two intact knee joints. Standardized cartilage plates (i.e., surface of 12.5 cm^2^) were crafted for the needs of the assay. The cartilage plates were secured in a gel mucoadhesion scaffold and were mounted on a Texture Analyzer TA.XT. Plus instrument (Tracomme, Schlieren, Switzerland). Volumes of 300 µL of undiluted hydrogel sample were dispensed onto the surface of the cartilage plate and were contained within the mucoadhesion ring. A 23-mm diameter steel gel mucoadhesion probe (Tracomme, Schlieren, Switzerland) was lowered onto the scaffold until contact was established with the cartilage plate surface, after travel through the hydrogel sample. A constant downward compression force of 0.5 N was then applied for 30 s, for the establishment of a stable system baseline state. Then, the mucoadhesion probe was automatically raised by the instrument at a constant detachment speed of 2 mm·s^−1^. The detachment force profile was recorded in triplicate for each hydrogel sample. A second run of measurements was then performed as described hereabove using the second type of cartilage sample. The peak detachment force (i.e., force of adhesion) and the work of adhesion were determined for each group.

### 4.5. Accelerated Hydrogel Degradation Assay

An in vitro oxidative challenge assay was used to assess the resistance of the considered commercial hydrogel preparations toward controlled accelerated degradation. Therefore, volumes of 250 µL of undiluted hydrogel sample were combined with 250 µL of undiluted hydrogen peroxide (i.e., H_2_O_2_ 30% *w*/*w*), simulating strong oxidative stress. The obtained samples were incubated at 37 °C for 30 min before endpoint analysis. An oscillatory rheology readout was used, with the same setup and settings as reported herein for the rheological behavior characterization assays. In order to obtain baseline values of G′ and G″ (i.e., non-challenged samples), volumes of 250 µL of undiluted hydrogel sample were combined with 250 µL of distilled water. After 30 min of incubation at 37 °C, the non-challenged samples were analyzed in oscillatory rheology. The residual fractions of the storage moduli and loss moduli, determined between the challenged and the non-challenged samples, were used to express the resistance of the system toward controlled oxidant-mediated degradation.

### 4.6. Benchmarking of Antioxidant Attributes for HA-Based Hydrogel Additives

The Trolox equivalent antioxidant capacity (TEAC) of mannitol and niacinamide samples was determined using a colorimetric Total Antioxidant Capacity Assay Kit, following the instructions of the manufacturer. Briefly, each sample was reconstituted in purified water at the same concentration as that used in the Ostenil Plus (i.e., 10 mg/mL mannitol) and Innoryos (i.e., 15 mg/mL niacinamide) products. Sample volumes of 20 μL were transferred to 96-well microtitration plates and volumes of 100 μL of the prepared reaction mix were added to each well. The plates were incubated at ambient temperature for 10 min. Absorbance values were determined at a wavelength of 570 nm on a Synergy Mx microplate reader (Biotek, Winooski, VT, USA) and the TEAC values were calculated based on an experimental Trolox standard curve. All assays were performed using six experimental replicates and the results were presented in absolute values of Trolox equivalents.

### 4.7. Statistical Analysis and Data Presentation

Data were reported as means accompanied by the corresponding standard deviations. For the statistical comparison of values from multi-group quantitative datasets, a one-way ANOVA test was performed, and it was followed by a post hoc Tukey’s multiple comparison test. A *p*-value < 0.05 was retained as a general base for statistical significance determination. Detailed levels of statistical significance can be found in the Results section and in the Supplementary Tables. The statistical calculations and/or data presentation were performed using Microsoft Excel (Microsoft Corporation, Redmond, WA, USA), Microsoft PowerPoint, and GraphPad Prism v. 8.0.2 (GraphPad Software, San Diego, CA, USA).

## Figures and Tables

**Table 1:** Comparative overview of the general attributes and characteristics of the considered HA-based commercial viscosupplementation products. The data used for the commercial hydrogel product technical comparison work were compiled from manufacturer-provided information. Each product has been widely clinically implemented for orthopedic use in OA patient treatment. HA, hyaluronic acid; MDa, megadalton; NA, non-applicable; OA, osteoarthritis.

Parameters	Investigated Commercial Hydrogel Products			
	Ostenil	Ostenil Plus	Synvisc	Innoryos
Manufacturer	TRB Chemedica; Geneva, Switzerland	TRB Chemedica; Geneva, Switzerland	Sanofi Genzyme; Cambridge, MA, USA	Albomed; Schwarzenbruck, Germany
Market Launch Year	1998	2009	1997	2022
Regulatory Classification	Class III device	Class III device	Class III device	Class III device
Specified Indications	Pain and mobility reduction in degenerative and traumatic affections of the knee and other synovial articulations	Pain and mobility reduction in degenerative and traumatic affections of the knee and other synovial articulations	Pain in OA of the knee in patients who have failed to respond adequately to conservative nonpharmacologic therapy and simple analgesics	Pain and decreased articular mobility associated with degenerative lesions of the knee and other synovial joints, including OA
HA Concentration/Polymer Type	10 mg/mL; Linear HA	20 mg/mL; Linear HA	8 mg/mL; Chemically cross-linked HA	22 mg/mL; Linear HA
HA Molecular Weight (Molecular Weight Class)	1.6 MDa (Intermediate)	1.6 MDa (Intermediate)	6 MDa (Hylan A; High)	1.2–2.2 MDa (Intermediate)
HA Sourcing	Biotechnology	Biotechnology	Avian	Biotechnology
Composition	1% HA; injectable buffer solution	2% HA; 1% mannitol; injectable buffer solution	0.8% Hylan G-F 20 ^1^; injectable buffer solution	2.2% HA; 1.5% niacinamide; injectable buffer solution
Quantities/Additives	NA	Mannitol	NA	Niacinamide ^2^
Volume/Unit	2.0 mL	2.0 mL	2.0 mL	2.0 mL
Administration Regimen	3–5 injections; 1 week interval	1–3 injections; 1 week interval	3 injections; 1 week interval	3 injections; 1 week interval

^1^ Hylan A (80%) and Hylan B (20%). ^2^ Niacinamide is also known as nicotinamide [32,33].

**Table 2:** Synoptic presentation of the various functional behaviors of the investigated commercial HA-based viscosupplementation products. Comparative operator gradings were attributed based solely on the obtained experimental results, taking into account and comparing each product’s performance to those of the other experimental groups. From the experimental data, it was possible to identify Innoryos as the overall best-performing product, from a multiparametric functional standpoint. HA, hyaluronic acid.

Functional Parameter	Overall Assessment/Comparative Operator Gradings ^1^			
	Ostenil	Ostenil Plus	Synvisc	Innoryos
Viscoelasticity	+	++	+++	+++
Lubricity	+	+	+++	+++
Bio-Adhesion	+	++	++	+++
Stability	–	++	++	+++

^1^ The comparative operator grading was performed by three experienced operators using the abbreviated nomenclature presented hereafter; (–) = unsatisfactory; (+) = sub-optimal; (++) = satisfactory; (+++) = optimal.

## Data Availability

The data presented in this study are available upon written and reasonable request from the corresponding authors.

## Supplementary Materials

The following supporting information can be downloaded at: https://www.mdpi.com/article/10.3390/gels9100808/s1. Figure S1: Complementary rheological data; Figure S2: Complementary tribological data; Figure S3: Inter-parameter correlation analyses; Figure S4: Complementary bio-adhesivity data; Figure S5: Complementary rheological data; Figure S6: Antioxidant capacity data; Table S1: Statistical analysis relative to rheological data; Table S2: Statistical analysis relative to tribological data; Table S3: Statistical analysis relative to tribological data; Table S4: Statistical analysis relative to bio-adhesion data; Table S5: Statistical analysis relative to bio-adhesion data; Table S6: Statistical analysis relative to stability data; Table S7: Statistical analysis relative to antioxidant activity data; Table S8: Functional parametric analysis possibilities for HA-based viscosupplementation hydrogel products.

## Abbreviations

ASTM	American Society for Testing and Materials
CE	European mark of conformity
Da	Daltons
η*	complex viscosity
EP	European Pharmacopoeia
FDA	US Food and Drug Administration
FRAP	ferric reducing antioxidant power
G′	storage modulus
G″	loss modulus
GAG	glycosaminoglycan
GPC	gel permeation chromatography
H_2_O_2_	hydrogen peroxide
HA	hyaluronic acid
HAS	hyaluronan synthases
HYAL	hyaluronidases
ISO	International Standards Organization
LVE	linear viscoelastic region
MD	medical device
MDa	megadalton
min	minute
MW	molecular weight
N	Newtons
NA	non-applicable
N·s	Newton seconds
ns	non-significant
OA	osteoarthritis
Pa	Pascals
Pa·s	Pascal seconds
PBS	phosphate-buffered saline
PDMS	polydimethylsiloxane
ROS	reactive oxygen species
SEC	size-exclusion chromatography
SF	synovial fluid
TEAC	Trolox equivalent antioxidant capacity
USA	United States of America
