# Supplementary material for: Ex Vivo Functional Benchmarking of Hyaluronan-Based Osteoarthritis Viscosupplement Products: Comprehensive Assessment of Rheological, Lubricative, Adhesive, and Stability Attributes
Source: Gels. 2023 Oct 9;9(10):808. doi: 10.3390/gels9100808 (PMC10606320; doi:10.3390/gels9100808)
Supplement: Supplementary file 1 [file gels-09-00808-s001.zip › gels-2631337-supplementary.pdf]

<u>Supplementary Materials</u>:

Ex Vivo Functional Benchmarking of Hyaluronan-Based Osteoarthritis Viscosupplement Products: Comprehensive Assessment of Rheological, Lubricative, Adhesive, and Stability Attributes

Alexandre Porcello, Farid Hadjab, Maryam Ajouaou, Virginie Philippe, Robin Martin, Philippe Abdel-Sayed, Nathalie Hirt-Burri, Corinne Scaletta, Wassim Raffoul, Lee Ann Applegate, Eric Allémann, Olivier Jordan and Alexis Laurent

1. Supplementary Figures

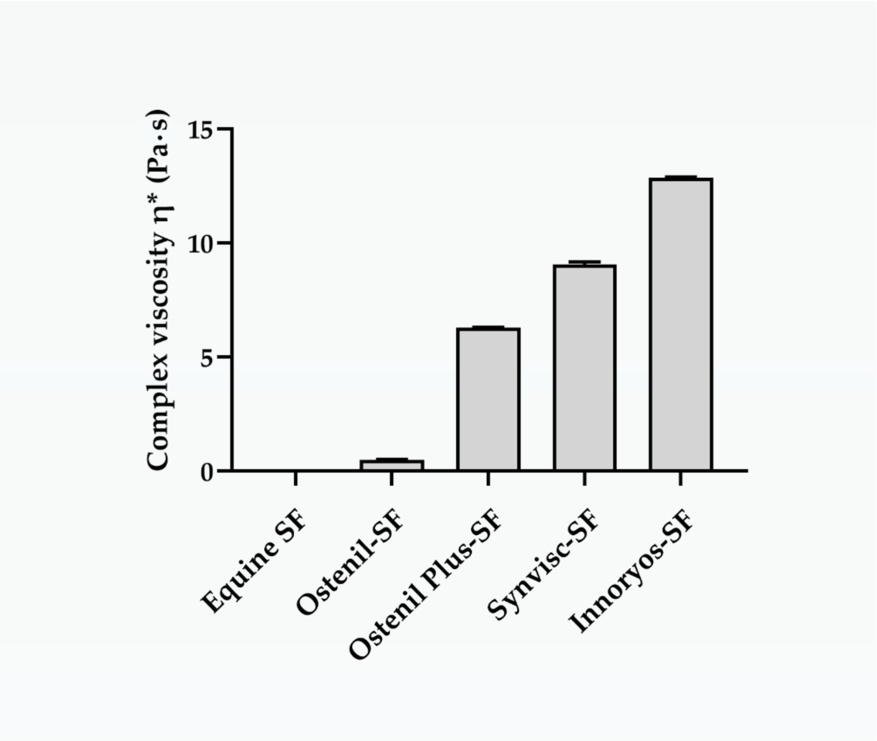

Figure S1. Results of ex vivo oscillatory rheology measurements for the four considered commercial hydrogel preparations, with comparative quantitative determination of the η* complex viscosity, in relation with the data presented in Figure 1. The samples were prepared by combination of the hydrogel with equal volumes of fresh equine SF and were analyzed at 22 °C with a frequency of 0.5 Hz, simulating a normal walking condition. Measurements were performed in triplicate and standard deviations were reported as error bars around mean values. All inter-group differences were found to be extremely statistically significant (*p*-value <0.0001). Detailed results of the statistical analysis are presented in Table S1. Pa·s, Pascal seconds; SF, synovial fluid.

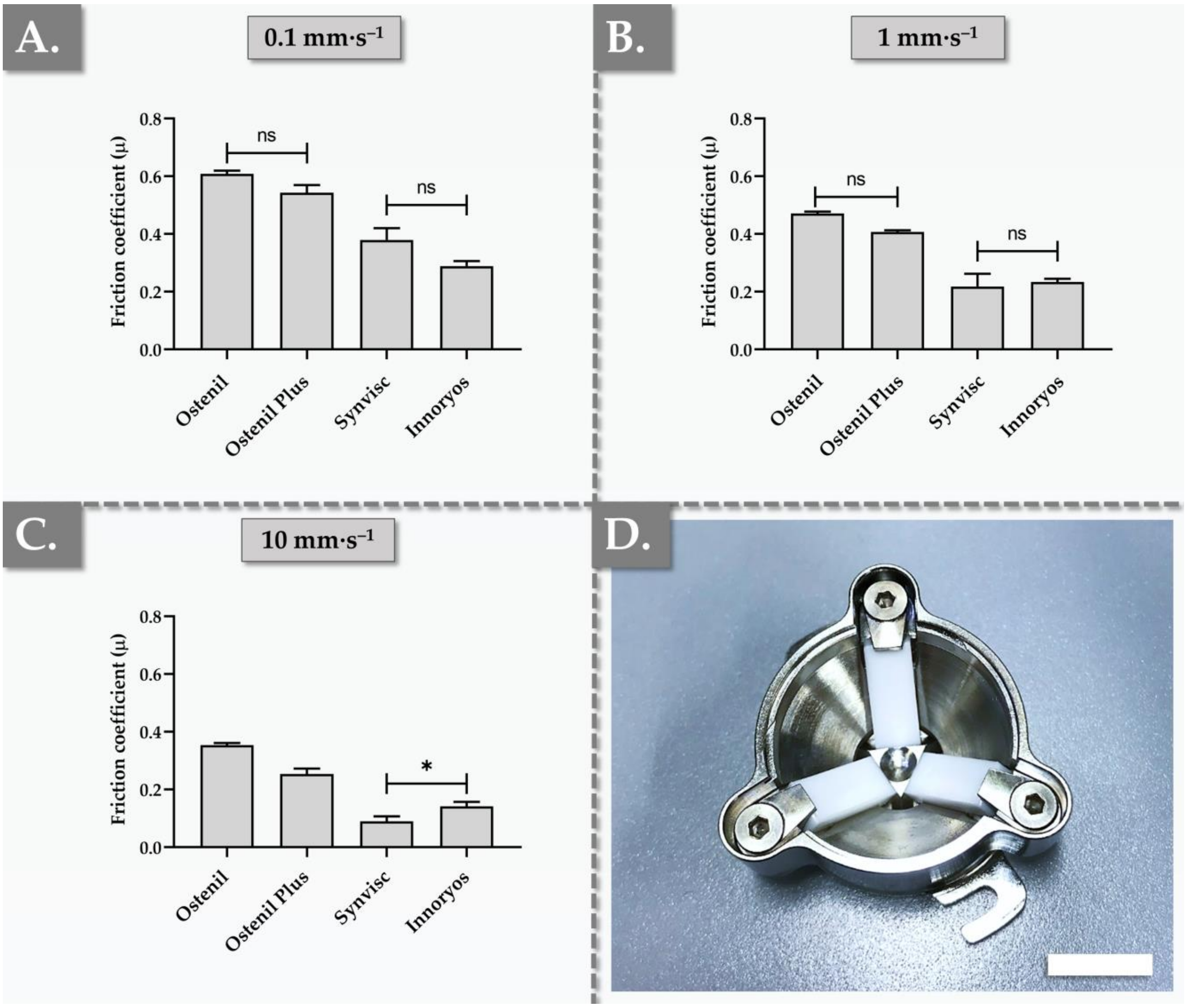

Figure S2. Results of in vitro rotational tribology measurements for the four considered commercial hydrogel preparations, using a tribology cell equipped with PDMS plates and a glass ball. The hydrogel samples were not diluted and were analyzed at 37 °C using various rotational sliding velocities. (**A**) Comparative quantitative determination of μ friction coefficients within the system under rotation at 0.1 $mm \cdot s^{-1}$. (**B**) Comparative quantitative determination of μ friction coefficients within the system under rotation at 1 $mm \cdot s^{-1}$. (**C**) Comparative quantitative determination of μ friction coefficients within the system under rotation at 10 $mm \cdot s^{-1}$. (**D**) In vitro rotational tribology setup, showing the lower portion of the tribology cell equipped with three PDMS plates. Scale bar = 10 mm. Measurements were performed in triplicate and standard deviations were reported as error bars around mean values. Statistically non-significant differences ("ns" or *p*-value >0.05) were highlighted as appropriate between the experimental groups. Statistically significant differences ("*" or *p*-value <0.05) were highlighted as appropriate between the experimental groups. Non-annotated inter-group differences were all found to be very statistically significant (*p*-value <0.01). Detailed results of the statistical analysis are presented in Table S3. ns, non-significant; PDMS, polydimethylsiloxane.

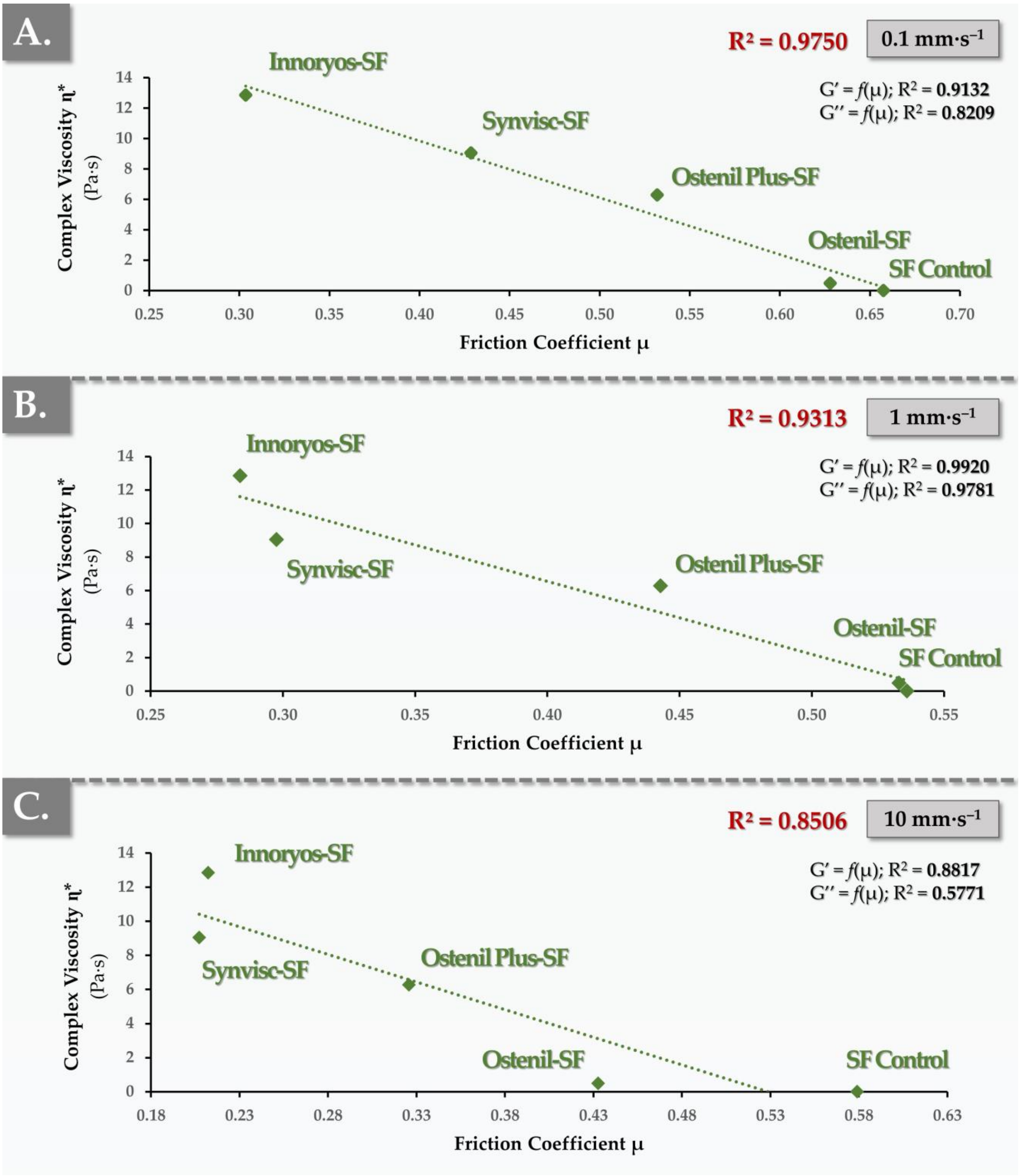

Figure S3. Results of the correlation analyses between the rheological and tribological attributes of the investigated hydrogel samples, in relation with the data presented in Figure 1, Figure 2, and Figure S1. (**A**) Correlation of μ friction coefficient values of each system (i.e., at low sliding velocity) with the η* complex viscosity values of the same system. (**B**) Correlation of μ friction coefficient values of each system (i.e., at intermediate sliding velocity) with the η* complex viscosity values of the same system. (**C**) Correlation of μ friction coefficient values of each system (i.e., at high sliding velocity) with the η* complex viscosity values of the same system. For each sliding velocity, the correlations between μ values and G′ values or G′′ values were determined and reported as the corresponding R^2 correlation coefficients. Pa·s, Pascal seconds; SF, synovial fluid.

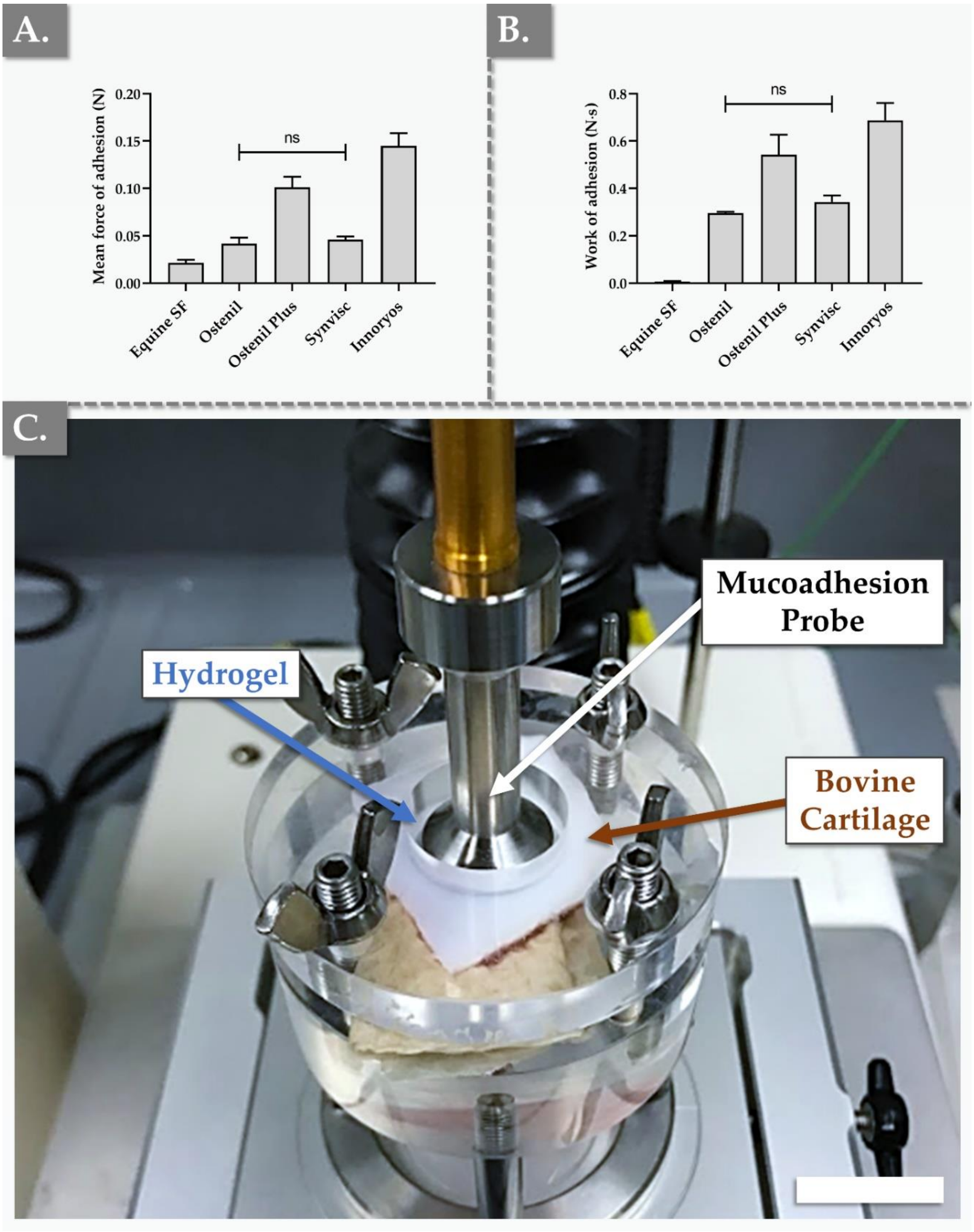

Figure S4. Results of ex vivo bio-adhesion measurements for the four considered commercial hydrogel preparations on bovine meniscal cartilage. The undiluted hydrogel samples were analyzed with a constant detachment speed of 2 mm·s^{-1} between a steel mucoadhesion probe and a fresh plane portion of cartilage. (**A**) Comparative quantitative determination of the peak force of adhesion values of the samples. (**B**) Comparative quantitative determination of the work of adhesion values of the samples. (**C**) Illustrative and annotated representation of the experimental ex vivo cartilage bio-adhesion setup. Scale bar = 30 mm. Measurements were performed in triplicate and standard deviations were reported as error bars around mean values. Non-annotated inter-group differences were all found to be very statistically significant (p-value <0.01). Detailed results of the statistical analysis are presented in Table S5. N, Newtons; N·s, Newton seconds; ns, non-significant; SF, synovial fluid.

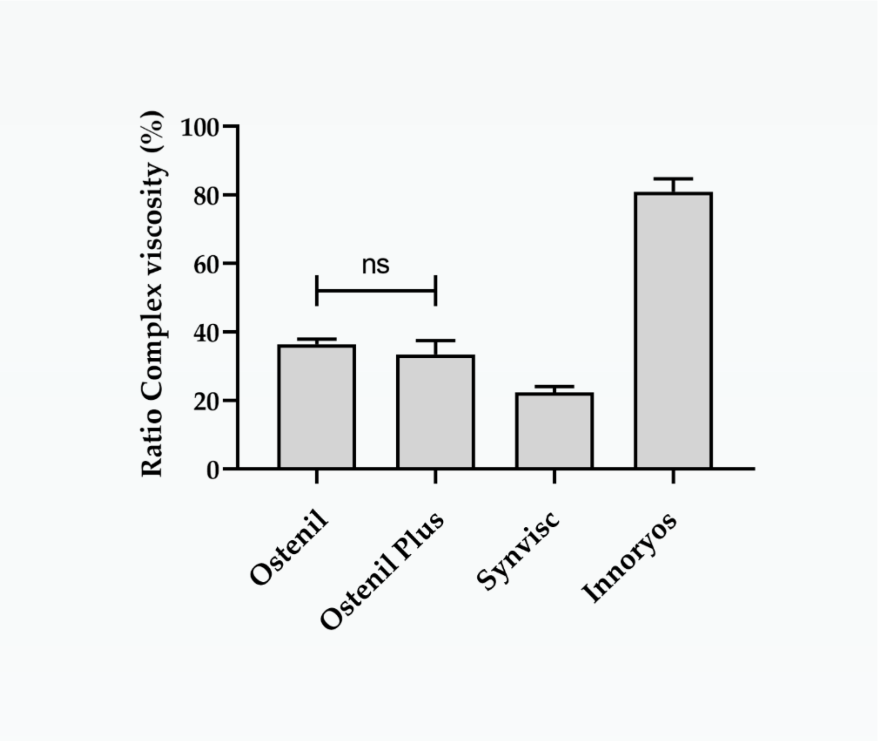

Figure S5. Results of accelerated stability studies, expressed as endpoint residual fractions of η* complex viscosity values for the four considered commercial hydrogel preparations, in relation with the data presented in Figure 4. The samples were prepared by exposure of the hydrogel to H_2O_2 for 30 min and were analyzed at 37 °C with a frequency of 0.5 Hz. Measurements were performed in triplicate and standard deviations were reported as error bars around mean values. Statistically non-significant differences ("ns" or *p*-value >0.05) were highlighted as appropriate between the experimental groups. Non-annotated inter-group differences were all found to be very statistically significant (*p*-value <0.01). Detailed results of the statistical analysis are presented in Table S6. min, minute; ns, non-significant.

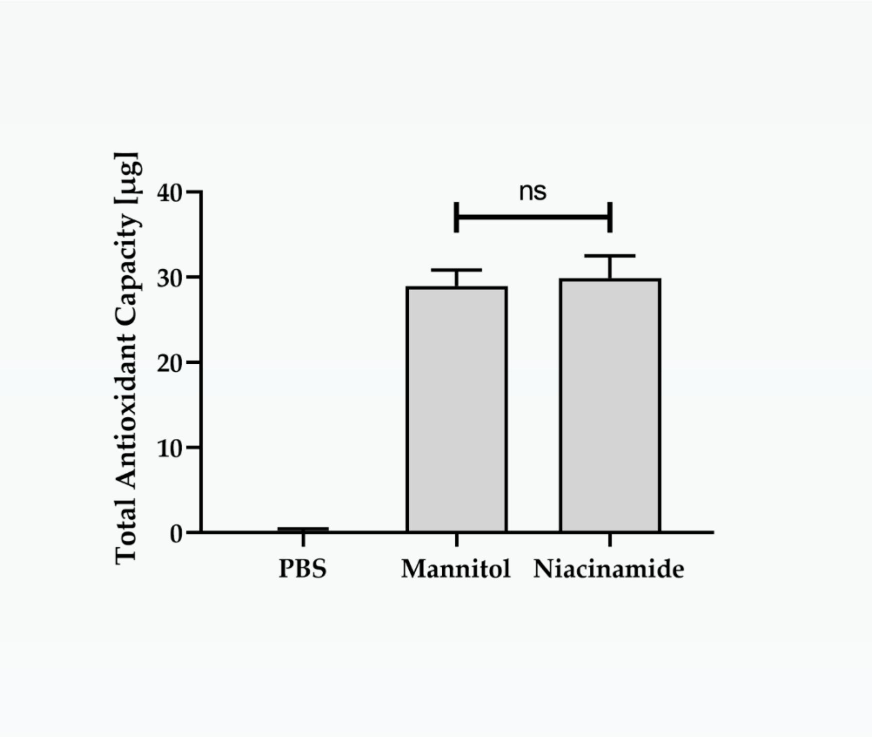

Figure S6. Experimental results of comparative intrinsic antioxidant capacity determination assays for mannitol and niacinamide. The results were expressed as the total equivalent antioxidant activity (i.e., equivalent μg of Trolox) of the samples, based on an experimental Trolox standard curve. Both excipients were characterized by antioxidant activity values which were significantly different from that of the PBS control group. Standard deviations were reported as error bars around mean values. Statistically non-significant differences ("ns" or *p*-value >0.05) were highlighted as appropriate between the experimental groups. Detailed results of the statistical analysis are presented in Table S7. ns, non-significant; PBS, phosphate-buffered saline.

2. Supplementary Tables

Table S1. Quantitative results of the post hoc Tukey's multiple comparison test, in relation with the comparative rheological data presented in Figure 1 (i.e., G′ storage moduli and G′′ loss moduli) and in Figure S1 (i.e., η* complex viscosity). Non-significant differences corresponded to a *p*-value > 0.05. ns, non-significant; Pa, Pascals; Pa·s, Pascal seconds; SF, synovial fluid.

Rheological Parameters	Compared Groups	Mean Absolute Difference (Pa or Pa·s)	Adjusted *p*-Value	Significance Level [1]
G′ Storage Modulus (Pa)	SF control / Ostenil-SF	–0.41	0.0299	*
	SF control / Ostenil Plus-SF	–12.52	< 0.0001	****
	SF control / Synvisc-SF	–26.79	< 0.0001	****
	SF control / Innoryos-SF	–27.52	< 0.0001	****
	Ostenil-SF / Ostenil Plus-SF	–12.11	< 0.0001	****
	Ostenil-SF / Synvisc-SF	–26.39	< 0.0001	****
	Ostenil-SF / Innoryos-SF	–27.12	< 0.0001	****
	Ostenil Plus-SF / Synvisc-SF	–14.27	< 0.0001	****
	Ostenil Plus-SF / Innoryos-SF	–15.00	< 0.0001	****
	Innoryos-SF / Synvisc-SF	0.73	0.0002	***
G′′ Loss Modulus (Pa)	SF control / Ostenil-SF	–1.45	< 0.0001	****
	SF control / Ostenil Plus-SF	–15.23	< 0.0001	****
	SF control / Synvisc-SF	–9.43	< 0.0001	****
	SF control / Innoryos-SF	–29.49	< 0.0001	****
	Ostenil-SF / Ostenil Plus-SF	–13.78	< 0.0001	****
	Ostenil-SF / Synvisc-SF	–7.98	< 0.0001	****
	Ostenil-SF / Innoryos-SF	–28.04	< 0.0001	****
	Ostenil Plus-SF / Synvisc-SF	5.80	< 0.0001	****
	Ostenil Plus-SF / Innoryos-SF	–14.26	< 0.0001	****
	Innoryos-SF / Synvisc-SF	20.07	< 0.0001	****
η* Complex Viscosity (Pa·s)	SF control / Ostenil-SF	–0.48	< 0.0001	****
	SF control / Ostenil Plus-SF	–6.28	< 0.0001	****
	SF control / Synvisc-SF	–9.04	< 0.0001	****
	SF control / Innoryos-SF	–12.84	< 0.0001	****
	Ostenil-SF / Ostenil Plus-SF	–5.80	< 0.0001	****
	Ostenil-SF / Synvisc-SF	–8.56	< 0.0001	****
	Ostenil-SF / Innoryos-SF	–12.36	< 0.0001	****
	Ostenil Plus-SF / Synvisc-SF	–2.76	< 0.0001	****
	Ostenil Plus-SF / Innoryos-SF	–6.57	< 0.0001	****
	Innoryos-SF / Synvisc-SF	3.80	< 0.0001	****

[1] A significance level described by one asterisk "*" corresponded to a *p*-value between 0.01 and 0.05. A significance level described by three asterisks "***" corresponded to a *p*-value between 0.0001 and 0.001. A significance level described by four asterisks "****" corresponded to a *p*-value inferior to 0.0001.

Table S2. Quantitative results of the post hoc Tukey's multiple comparison test, in relation with the comparative tribological data presented in Figure 2 (i.e., ex vivo tribology setup). Non-significant ("ns") differences corresponded to a *p*-value > 0.05. ns, non-significant; SF, synovial fluid.

Rotational Speed Settings	Compared Groups	Mean Absolute Difference in μ Friction Coefficient	Adjusted *p*-Value	Significance Level [1]
Low Sliding Velocity (0.1 mm·s^{-1})	SF control / Ostenil-SF	0.03	0.8985	ns
	SF control / Ostenil Plus-SF	0.13	0.0140	*
	SF control / Synvisc-SF	0.23	< 0.0001	****
	SF control / Innoryos-SF	0.35	< 0.0001	****
	Ostenil-SF / Ostenil Plus-SF	0.10	0.0745	ns
	Ostenil-SF / Synvisc-SF	0.20	0.0002	***
	Ostenil-SF / Innoryos-SF	0.32	< 0.0001	****
	Ostenil Plus-SF / Synvisc-SF	0.10	0.0501	ns
	Ostenil Plus-SF / Innoryos-SF	0.23	< 0.0001	****
	Innoryos-SF / Synvisc-SF	–0.13	0.0145	*
Intermediate Sliding Velocity (1 mm·s^{-1})	SF control / Ostenil-SF	0.01	> 0.9999	ns
	SF control / Ostenil Plus-SF	0.09	0.1003	ns
	SF control / Synvisc-SF	0.24	< 0.0001	****
	SF control / Innoryos-SF	0.25	< 0.0001	****
	Ostenil-SF / Ostenil Plus-SF	0.09	0.1168	ns
	Ostenil-SF / Synvisc-SF	0.24	< 0.0001	****
	Ostenil-SF / Innoryos-SF	0.25	< 0.0001	****
	Ostenil Plus-SF / Synvisc-SF	0.15	0.0057	**
	Ostenil Plus-SF / Innoryos-SF	0.16	0.0027	**
	Innoryos-SF / Synvisc-SF	–0.01	0.9940	ns
High Sliding Velocity (10 mm·s^{-1})	SF control / Ostenil-SF	0.15	0.0001	***
	SF control / Ostenil Plus-SF	0.25	< 0.0001	****
	SF control / Synvisc-SF	0.37	< 0.0001	****
	SF control / Innoryos-SF	0.37	< 0.0001	****
	Ostenil-SF / Ostenil Plus-SF	0.11	0.0026	**
	Ostenil-SF / Synvisc-SF	0.23	< 0.0001	****
	Ostenil-SF / Innoryos-SF	0.22	< 0.0001	****
	Ostenil Plus-SF / Synvisc-SF	0.12	0.0010	**
	Ostenil Plus-SF / Innoryos-SF	0.11	0.0015	**
	Innoryos-SF / Synvisc-SF	0.01	0.9995	ns

[1] A significance level described by one asterisk "*" corresponded to a *p*-value between 0.01 and 0.05. A significance level described by two asterisks "**" corresponded to a *p*-value between 0.001 and 0.01. A significance level described by three asterisks "***" corresponded to a *p*-value between 0.0001 and 0.001. A significance level described by four asterisks "****" corresponded to a *p*-value inferior to 0.0001.

Table S3. Quantitative results of the post hoc Tukey's multiple comparison test, in relation with the comparative tribological data presented in Figure S2 (i.e., in vitro tribology setup). Non-significant ("ns") differences corresponded to a *p*-value > 0.05. ns, non-significant; SF, synovial fluid.

Rotational Speed Settings	Compared Groups	Mean Absolute Difference in μ Friction Coefficient	Adjusted *p*-Value	Significance Level [1]
Low Sliding Velocity (0.1 $mm \cdot s^{-1}$)	SF control / Ostenil	0.0490	0.6284	ns
	SF control / Ostenil Plus	0.1147	0.0326	*
	SF control / Synvisc	0.2781	< 0.0001	****
	SF control / Innoryos	0.3682	< 0.0001	****
	Ostenil / Ostenil Plus	0.0658	0.3581	ns
	Ostenil / Synvisc	0.2292	< 0.0001	****
	Ostenil / Innoryos	0.3193	< 0.0001	****
	Ostenil Plus / Synvisc	0.1634	0.0022	**
	Ostenil Plus / Innoryos	0.2535	< 0.0001	****
	Innoryos / Synvisc	–0.0901	0.1200	ns
Intermediate Sliding Velocity (1 $mm \cdot s^{-1}$)	SF control / Ostenil	0.0646	0.2534	ns
	SF control / Ostenil Plus	0.1291	0.0050	**
	SF control / Synvisc	0.3175	< 0.0001	****
	SF control / Innoryos	0.3019	< 0.0001	****
	Ostenil / Ostenil Plus	0.0645	0.2548	ns
	Ostenil / Synvisc	0.2529	< 0.0001	****
	Ostenil / Innoryos	0.2373	< 0.0001	****
	Ostenil Plus / Synvisc	0.1884	0.0001	***
	Ostenil Plus / Innoryos	0.1728	0.0003	***
	Innoryos / Synvisc	0.0156	0.9843	ns
High Sliding Velocity (10 $mm \cdot s^{-1}$)	SF control / Ostenil	0.2203	< 0.0001	****
	SF control / Ostenil Plus	0.3212	< 0.0001	****
	SF control / Synvisc	0.4839	< 0.0001	****
	SF control / Innoryos	0.4328	< 0.0001	****
	Ostenil / Ostenil Plus	0.1009	0.0001	***
	Ostenil / Synvisc	0.2636	< 0.0001	****
	Ostenil / Innoryos	0.2125	< 0.0001	****
	Ostenil Plus / Synvisc	0.1627	< 0.0001	****
	Ostenil Plus / Innoryos	0.1116	< 0.0001	****
	Innoryos / Synvisc	0.0511	0.0442	*

[1] A significance level described by one asterisk "*" corresponded to a *p*-value between 0.01 and 0.05. A significance level described by two asterisks "**" corresponded to a *p*-value between 0.001 and 0.01. A significance level described by three asterisks "***" corresponded to a *p*-value between 0.0001 and 0.001. A significance level described by four asterisks "****" corresponded to a *p*-value inferior to 0.0001.

Table S4. Quantitative results of the post hoc Tukey's multiple comparison test, in relation with the comparative ex vivo bio-adhesion data presented in Figure 3 (i.e., tibial cartilage setup). Non-significant ("ns") differences corresponded to a *p*-value > 0.05. N, Newtons; N·s, Newton seconds; ns, non-significant; SF, synovial fluid.

**Bio-Adhesion Parameters**	**Compared Groups**	**Mean Absolute Difference** (N or N·s)	**Adjusted *p*-Value**	**Significance Level** [1]
Force of Adhesion (N)	SF control / Ostenil	–0.10	< 0.0001	****
	SF control / Ostenil Plus	–0.25	< 0.0001	****
	SF control / Synvisc	–0.22	< 0.0001	****
	SF control / Innoryos	–0.49	< 0.0001	****
	Ostenil / Ostenil Plus	–0.15	< 0.0001	****
	Ostenil / Synvisc	–0.12	< 0.0001	****
	Ostenil / Innoryos	–0.39	< 0.0001	****
	Ostenil Plus / Synvisc	0.03	0.1338	ns
	Ostenil Plus / Innoryos	–0.24	< 0.0001	****
	Innoryos / Synvisc	0.27	< 0.0001	****
Work of Adhesion (N·s)	SF control / Ostenil	–0.06	0.0038	**
	SF control / Ostenil Plus	–0.21	< 0.0001	****
	SF control / Synvisc	–0.16	< 0.0001	****
	SF control / Innoryos	–0.62	< 0.0001	****
	Ostenil / Ostenil Plus	–0.15	< 0.0001	****
	Ostenil / Synvisc	–0.10	< 0.0001	****
	Ostenil / Innoryos	–0.56	< 0.0001	****
	Ostenil Plus / Synvisc	0.05	0.0066	**
	Ostenil Plus / Innoryos	–0.41	< 0.0001	****
	Innoryos / Synvisc	0.46	< 0.0001	****

[1] A significance level described by two asterisks "**" corresponded to a *p*-value between 0.001 and 0.01. A significance level described by four asterisks "****" corresponded to a *p*-value inferior to 0.0001.

Table S5. Quantitative results of the post hoc Tukey's multiple comparison test, in relation with the comparative ex vivo bio-adhesion data presented in Figure S4 (i.e., meniscal cartilage setup). Non-significant ("ns") differences corresponded to a *p*-value > 0.05. N, Newtons; N·s, Newton seconds; ns, non-significant; SF, synovial fluid.

Bio-Adhesion Parameters	Compared Groups	Mean Absolute Difference (N or N·s)	Adjusted *p*-Value	Significance Level [1]
Force of Adhesion (N)	SF control / Ostenil	–0.0203	0.0891	ns
	SF control / Ostenil Plus	–0.0797	< 0.0001	****
	SF control / Synvisc	–0.0245	0.0351	*
	SF control / Innoryos	–0.1233	< 0.0001	****
	Ostenil / Ostenil Plus	–0.0594	< 0.0001	****
	Ostenil / Synvisc	–0.0042	0.9714	ns
	Ostenil / Innoryos	–0.1030	< 0.0001	****
	Ostenil Plus / Synvisc	0.0552	< 0.0001	****
	Ostenil Plus / Innoryos	–0.0437	0.0007	***
	Innoryos / Synvisc	0.0988	< 0.0001	****
Work of Adhesion (N·s)	SF control / Ostenil	–0.2898	0.0003	***
	SF control / Ostenil Plus	–0.5356	< 0.0001	****
	SF control / Synvisc	–0.3366	< 0.0001	****
	SF control / Innoryos	–0.6816	< 0.0001	****
	Ostenil / Ostenil Plus	–0.2458	0.0012	**
	Ostenil / Synvisc	–0.0469	0.7965	ns
	Ostenil / Innoryos	–0.3918	< 0.0001	****
	Ostenil Plus / Synvisc	0.1990	0.0056	**
	Ostenil Plus / Innoryos	–0.1459	0.0381	*
	Innoryos / Synvisc	0.3449	< 0.0001	****

[1] A significance level described by two asterisks "**" corresponded to a *p*-value between 0.001 and 0.01. A significance level described by four asterisks "****" corresponded to a *p*-value inferior to 0.0001.

Table S6. Quantitative results of the post hoc Tukey's multiple comparison test, in relation with the in vitro rheological data presented in Figure 4 (i.e., G′ storage moduli and G′′ loss moduli) and in Figure S5 (i.e., η* complex viscosity). Non-significant ("ns") differences corresponded to a *p*-value > 0.05. ns, non-significant; SF, synovial fluid.

Rheological Parameters	Compared Groups	Mean Absolute Difference (%)	Adjusted *p*-Value	Significance Level [1]
Residual Fraction of G′ Storage Modulus (%)	Ostenil / Ostenil Plus	−14.91	0.0012	**
	Ostenil / Synvisc	−14.31	0.0016	**
	Ostenil / Innoryos	−65.26	< 0.0001	****
	Ostenil Plus / Synvisc	0.60	0.9943	ns
	Ostenil Plus / Innoryos	−50.36	< 0.0001	****
	Innoryos / Synvisc	50.95	< 0.0001	****
Residual Fraction of G′′ Loss Modulus (%)	Ostenil / Ostenil Plus	−7.87	0.0258	*
	Ostenil / Synvisc	−14.22	0.0007	***
	Ostenil / Innoryos	−54.03	< 0.0001	****
	Ostenil Plus / Synvisc	−6.34	0.0700	ns
	Ostenil Plus / Innoryos	−46.16	< 0.0001	****
	Innoryos / Synvisc	39.82	< 0.0001	****
Residual Fraction of η* Complex Viscosity (%)	Ostenil / Ostenil Plus	3.02	0.6240	ns
	Ostenil / Synvisc	14.08	0.0019	**
	Ostenil / Innoryos	−44.46	< 0.0001	****
	Ostenil Plus / Synvisc	11.06	0.0084	**
	Ostenil Plus / Innoryos	−47.49	< 0.0001	****
	Innoryos / Synvisc	58.55	< 0.0001	****

[1] A significance level described by one asterisk "*" corresponded to a *p*-value between 0.01 and 0.05. A significance level described by two asterisks "**" corresponded to a *p*-value between 0.001 and 0.01. A significance level described by three asterisks "***" corresponded to a *p*-value between 0.0001 and 0.001. A significance level described by four asterisks "****" corresponded to a *p*-value inferior to 0.0001.

Table S7. Quantitative results of the post hoc Tukey's multiple comparison test, in relation with the antioxidant activity data presented in Figure S6. Non-significant ("ns") differences corresponded to a p-value > 0.05. ns, non-significant; PBS, phosphate-buffered saline.

**Compared Groups**	**Mean Absolute Difference** (µg Trolox)	**Adjusted *p*-Value**	**Significance Level** [1]
PBS / Mannitol	–28.77	< 0.0001	****
PBS / Niacinamide	–29.71	< 0.0001	****
Mannitol / Niacinamide	–0.94	0.7850	ns

[1] A significance level described by four asterisks "****" corresponded to a p-value inferior to 0.0001.

Table S8. Description of HA-based viscosupplementation product parameters and functional attributes with identified relevance within the ad hoc developmental process. It is to note that the specified reference documents and the related technical options are not exhaustively listed, yet the contents of the table constitute a comprehensive overview. ASTM, American Society for Testing and Materials; EP, European Pharmacopoeia; FRAP, ferric reducing antioxidant power; GPC, gel permeation chromatography; HA, hyaluronic acid; HYAL, hyaluronidases; ISO, International Standards Organization; NA, non-applicable; ROS, reactive oxygen species; SEC, size-exclusion chromatography; SF, synovial fluid.

Product Parameters / Functional Attributes	Appropriate Analytical Methods	Applicable Norms / Standards / Guidelines	Remarks / Technical Options	Level of Importance within the Developmental Process
Viscosity	Rotational rheology; Viscometry	Pharmacopoeia (e.g., EP chapter 2.2.10); ASTM (e.g., ASTM D445); ISO (e.g., ISO 3219)	± SF	HIGH
Storage Modulus	Oscillatory rheology	ASTM (e.g., ASTM D4440-15); ISO (e.g., ISO 3219-2:2021)	At a frequency of 0.5 Hz or 2.5 Hz; ± SF	HIGH
Loss Modulus	Oscillatory rheology	ASTM (e.g., ASTM D4440-15); ISO (e.g., ISO 3219-2:2021)	At a frequency of 0.5 Hz or 2.5 Hz; ± SF	MODERATE
Complex Viscosity	Oscillatory rheology	ASTM (e.g., ASTM D4440-15); ISO (e.g., ISO 3219-2:2021)	At a frequency of 0.5 Hz or 2.5 Hz; ± SF	HIGH
Coefficient of Friction	Tribology; Atomic force microscopy	ASTM (e.g., ASTM G99-17)	± cartilage explant; ± SF	HIGH
Cohesivity	Drop weight method; Five-point grading scale; Spreadability test	NA	Qualitative & quantitative methods available; Use of 2 methods is advised	LOW

Injectability	Injection force measurement; Syringeability	ISO (e.g., ISO 11608)	With appropriate syringe; Influence of the needle gauge & length	**LOW**
(Bio)-Adhesion	Probe tack test; Peeling test; Rolling test	ASTM (e.g., ASTM D2979; ASTM F2255-05)	± cartilage explant	**MODERATE**
Antioxidant Capacity	DPPH assay; Trolox assay; FRAP assay; Total phenolic content; Cell-based antioxidant assay	ISO (e.g., ISO 14502-1:2005)	Use of 2 orthogonal methods is advised; Assay types include spectrophotometric, electrochemical & chromatography techniques	**MODERATE**
Resistance to HYAL	HYAL challenge test with various readouts (e.g., rheology, tribology, colorimetry, gravimetry, SEC, GPC)	NA	Choice of appropriate hyaluronidase source is important; Assay duration depends on enzyme concentrations	**HIGH**
Resistance to ROS	ROS challenge test with various readouts (e.g., rheology, tribology, colorimetry, gravimetry, SEC, GPC)	NA	Choice of appropriate oxidant source (e.g., H_2O_2) is important; Assay duration depends on oxidant concentrations	**HIGH**
Resistance Against Wear	Tribology (e.g., pin on disc test, Fretting Wear Test) & evaluation with gravimetry, size analysis, or microscopy	ASTM (e.g., ASTM G99); ISO (e.g., ISO 14243-1)	± cartilage explant	**HIGH**
Swelling Ratio	Gravimetry	NA	Choice of water or physiological solution as swelling medium	**LOW**
